# Sulfonamide resistance gene *sul4* is hosted by common wastewater sludge bacteria and found in various newly described contexts and hosts

**DOI:** 10.1128/spectrum.00857-25

**Published:** 2025-11-19

**Authors:** Melina Markkanen, Denise Pezzutto, Marko Virta, Antti Karkman

**Affiliations:** 1 Department of Microbiology, University of Helsinki 3835, Helsinki, Finland; 2 Multidisciplinary Center of Excellence in Antimicrobial Resistance Research, University of Helsinki 3835, Helsinki, Finland; Kwame Nkrumah University of Science and Technology College of Health Sciences, Kumasi, Ghana

**Keywords:** antibiotic resistance, wastewater, sludge, *sul4*, sulfonamide resistance, bacterial methylation, metagenome-assembled genome, integrons, insertion sequences, IS*CR28*

## Abstract

The introduction of the first broad-spectrum antibiotics, sulfonamide drugs, fundamentally revolutionized medicine in the 1930s. Shortly after, and ever since, sulfonamide resistance genes (*sul* genes) have been widely detected. Still, the most recent variant of these genes, *sul4*, was first described only in 2017, and its host range and transmission mechanisms are still largely unknown. Here, we applied PacBio long-read metagenomic sequencing and bacterial methylation signals to investigate the genetic contexts and bacterial carriage of the *sul4* gene in wastewater. Furthermore, we extended our description of *sul4* carriers to previously published data sets. Our results indicate that *sul4* is prominently found in sludge and hosted by various bacteria, such as the species from the phyla Myxococcota and Chloroflexota and genera *Trichlorobacter* and *Desulfobacillus*, which are commonly found in activated sludge. Additionally, according to our results, *sul4* has already spread into multiple strains of opportunistic human pathogens, such as *Aeromonas* and *Moraxella,* in addition to the previously described *Salmonella*. The sequence region flanking *sul4* included a truncated *fol*K gene and an IS*CR28* element and exhibited a high degree of conservation across the investigated sequences. Furthermore, the module was associated with various integron integrase genes. Also, other mobility-related elements that could further increase the likelihood of *sul4* mobilization were detected. Altogether, our results describing the *sul4* hosts of bacteria from distant lineages indicate the efficient mobility of *sul4* by genetic elements that traverse both clinical and environmental bacteria. Finally, we suggest that wastewater may provide favorable conditions for such horizontal gene transfer events.

**IMPORTANCE:**

Antibiotic resistance is an ancient phenomenon and a common trait of many environmental bacteria. However, human activities in the post-antibiotic era, coupled with the bacteria’s ability to exchange genetic material across different lineages, have drastically increased the spread of resistance traits among bacteria from various niches. The primary concern is the resistance genes encoded by infections causing pathogens, already causing over 1 million deaths annually and indirectly contributing to nearly 4 million more. Therefore, understanding the bacteria that harbor ARGs and the genetic mechanisms driving their mobilization is crucial for understanding the dynamics and emerging trends of resistance. Here, we focus on revealing these crucial aspects of the newly discovered sulfonamide resistance gene, *sul4*. Given the limitations of the metagenomic approach in linking the functional genes to their host genomes, the significance of our research lies in our workflow, which allows this linkage through the identification of shared methylation profiles.

## INTRODUCTION

Increased antibiotic resistance threatens our current dependence on effective antibiotics in medicine, animal husbandry, and food production systems (1, 2). Besides the accumulation of point mutations in bacterial DNA, resistance may emerge by horizontal transfer of antibiotic resistance genes (ARGs) mediated by mobile genetic elements (3), such as plasmids (4, 5), IS elements (6), and resistance gene cassettes encoded by integrons (7). Integrons are genetic elements that capture and express genes as cassettes obtained via site-specific recombination at *attI* and *attC* sites and mediated by an integrase gene (7). IS*CR* (insertion sequence common regions) are a recently discovered and expanding group of IS*91*-like transposable elements that can translocate adjacent genes, such as ARGs, via a rolling-circle mechanism by *ori*IS (origin of replication) and *ter*IS (replication terminator) terminal sequences (8, 9). Prophage-mediated spread of ARGs has also been recognized, though its importance requires further investigation (10, 11). The transfer of ARGs carried by environmental bacteria into clinically significant bacterial hosts has been accelerated by human activities during the past decades (12, 13). Therefore, besides monitoring the occurrence of known resistance genes and identifying newly emerging ARGs, it is crucially important to study their surrounding genetic context and the bacteria that carry them. This aids in assessing their potential as high-risk ARGs that could migrate to new hosts and contexts (3, 14).

In 2017, Razavi and colleagues made the first discovery of the novel gene *sul4*, which confers resistance to sulfonamide antibiotics (15). This finding came decades after the identification of earlier *sul* gene variants: *sul1* in 1975 (16), *sul2* in 1980 (17), and *sul3* in 2003 (18). Ever since the introduction of sulfonamide antibiotics in 1935, they have been widely used in human medicine (19). Moreover, they are among the five most-used antibiotics in veterinary medicine for treating livestock diseases, such as gastrointestinal and respiratory infections, and, in some cases, as growth promoters (1) and specifically more used in many low- and middle-income countries (20, 21) due to their affordability. Sulfonamides act by competing with the precursor of dihydropteroate (DHP) synthase, p-aminobenzoic acid, which is crucial for folate biosynthesis. Folate pathway inhibition, in turn, blocks the bacterial purine synthesis for DNA production (19). The modified DHP synthases encoded by *sul* genes bypass the inhibition caused by sulfonamides, enabling resistance to these antibiotics (22).

Sul4 shares only 34% amino acid identity with DHP synthases and confers high-level resistance to sulfonamides in *Escherichia coli* transconjugants (15), even with one amino acid substitution (23). The near genetic context of *sul4,* including integron integrase, a truncated *fol*K gene related to the folate biosynthesis pathway, and the IS*CR* element, has already been confirmed by at least two studies (15, 23). However, the broader context and host range of *sul4* remain unclear because the initial discovery of the gene, as well as the subsequent retrospective surveys, relied on culture-independent methods (15, 24, 25). To our knowledge, the first, and currently only, description of *sul4* with confirmed sulfonamide resistance phenotype in a known bacterial host species was in a multidrug-resistant isolate of *Salmonella enterica* in 2023 (23). This discovery provides insight into the *sul4* gene’s potential to spread among clinically significant strains.

Despite their extensive use over the decades, new sequence variants of *sul* genes have emerged rarely, especially compared to beta-lactamase class ARGs. However, the latest observations of *sul4* suggest that there is a selection pressure for this gene to emerge and spread around the world (15, 23–25). Still, the variety of the hosts and mobility mechanisms must be investigated to be able to understand the risk posed by *sul4* for the efficacy of sulfonamide antibiotics. In this study, we aimed to reveal the different host bacteria and genetic contexts of *sul4* found in influent and effluent wastewater as well as in dried sludge. Wastewater collects fecal material and gut microbiota from entire populations linked to the sewer system. Furthermore, the communal wastewater resistome, which encompasses all detected ARGs, seems to reflect the resistance patterns observed in clinical reports and capture the spatio-temporal variations in resistance trends across populations (26–28).

Different compartments of wastewater treatment plants (WWTPs) show differences in the diversity and composition of resistomes, and this is linked to the changes in the microbiomes (29, 30). Activated sludge holds a central role in wastewater treatment, as it enables the microorganisms to work for nutrient removal through different biochemical processes (22). Contradictory results have been presented regarding the removal versus enrichment of ARGs and, for instance, integron integrase-encoding genes during the treatment process (24, 29, 31). However, these genes are transcribed more prominently in the WWTP outlet than in the incoming wastewater, reflecting bacterial responses to environmental stressors such as antimicrobial compounds and rapidly changing conditions during the treatment process (30). While an effective wastewater treatment system removes most bacteria and the ARGs with them, some ARGs may become enriched during the process due to the proliferation of their host bacteria (29, 31) and even end up in the WWTP discharge (32).

Although metagenomic sequencing in wastewater detects a wide variety of ARGs, this method, particularly short-read sequencing, often fails to reveal how these genes are carried, transmitted, or their origin. Yet, this information, corresponding to each ARG’s risk and significance in the overall resistance burden, is crucial, especially in the case of novel emerging ARGs (4, 33). Connecting functional genes, such as ARGs, and mobile genetic elements to their carrier bacteria remains a challenge of the metagenomic approach (34, 35). However, advancements in long-read sequencing technologies by PacBio and Nanopore, along with improved data analysis methods, have begun to enable the investigation of extended contiguous genetic contexts (36, 37). Here, we used PacBio long-read metagenomic sequencing, with read lengths extending well over typical ARG lengths to enable reliable investigation of *sul4* gene contexts. Additionally, we took advantage of the unique bacterial methylation patterns to resolve the bacterial hosts for *sul4* in the complex wastewater microbial communities. DNA methylation, catalyzed by MTases, is the most important epigenetic modification mechanism in bacteria (36). Due to the bacterial differences in MTase presence, species-specific methylation profiles have been used to resolve genetic material of shared origin before (38). Current algorithms for binning metagenome-assembled genomes (MAGs) using sequence composition and differential coverage may struggle to detect strain-level differences or, for example, link plasmid sequences with varying coverages to their associated host genomes (38). Applying methylation signals to link contigs together according to their source organism can help overcome these limitations.

## MATERIALS AND METHODS

### Sample collection

Composite samples from influent and effluent wastewater and dried sludge were collected on 3 days in 2019 (18, 20, and 26 March 2019) at one of the two wastewater treatment plants responsible for processing wastewater in the Helsinki metropolitan area in Finland. The population of this area is about 1.6 million. In 2019, the sampled WWTP received 107 million cubic meters of influent wastewater, accounting for 71.8% of the total influent volume in the Helsinki metropolitan area. This was due to the other WWTP being phased out and replaced by a new facility in the following years. The sampled treatment plant received wastewater from households and industry. In 2019, the latter made up approximately 5% of the influent when counting together both WWTPs at the Helsinki metropolitan area. Rainwater runoff is also directed to the WWTP. In the sampled WWTP, nearly 65,000 kg of sludge was produced by the treatment process in 2019. Ninety-three percent of the dried sludge was delivered to several companies for further processing by composting-based methods, ultimately resulting in products suitable for agricultural or landscaping use. Part of the sludge composting runoff was pumped back to the WWTP. Samples were collected during a period when wastewater temperatures ranged between 8 and 11.5°C, which represented the lowest annual temperatures at the WWTP due to cold rainwater and snowmelt runoff partially entering the sewage system during the sampling period (39). In 2019, the yearly average of pH of the dried sludge was 8.2 (39).

### DNA extraction, sequencing, and preliminary read processing

Wastewater samples were filtered through a 0.22 µm filter, and DNA was extracted from the filter. The Qiagen DNeasy PowerWater kit was used with bead beating for extracting the DNA, which was sent to the Institute of Biotechnology, University of Helsinki, for library preparation and PacBio long-read metagenomic sequencing. A PacBio Sequel II instrument was used for Single Molecule Real-Time (SMRT) sequencing with 12 SMRT cells. High-fidelity reads with kinetics information were prepared using PacBio BAM toolkit programs (https://github.com/PacificBiosciences/pbtk) to allow methylation detection, while reads without the tags were used for metagenomic assembly. Additionally, paired-end short reads were obtained using Illumina NovaSeq. Short-read data were used for read recruitment for MAGs and the bacterial diversity analysis by MetaPhlAn4 (40). These data were analyzed and visualized using phyloseq (41), vegan (42), and ggplot2 (43) in R (v.4.4.0) (44) and RStudio (v.2024.12.0+467.pro1) (45) (Supplemental Methods).

### Metagenomic assembly and identification of *sul4* reads and contigs

To improve the reliability of the assemblies, two assemblers were used for the metagenomic assembly of the wastewater samples, hifiasm-meta (v.0.18.0) (46) and metaFlye (v.2.9.3) (47). The first mentioned was run with the default options, and the latter with the additional parameter --min-overlap 4000. Reads encoding *sul4* were extracted using a custom Snakemake (48) workflow, which included steps for mapping the reads to an indexed *sul4* reference using pbmm2 align (v.1.9.0) (https://github.com/PacificBiosciences/pbmm2) and converting the mapped reads from BAM to FASTA files with bam2fasta (v.1.0.0) (https://github.com/PacificBiosciences/pbtk). The *sul4* contigs were extracted from the sample assemblies using BLAST (49).

### Collection of *sul4* host sequences from public databases

The *sul4* reference gene (NG_056174.1) was used to explore the matching sequences in the National Center for Biotechnology Information (NCBI) and Integrated Microbial Genomes and Microbiomes (IMG) databases. Sequences with an alignment length greater than 500 bp, relative to the reference gene (864 bp), were included in the downstream analysis, yielding 11 hits. Data collection was completed by 30 April 2024. Sequence similarity among established *sul* genes, distantly *sul4*-resembling genes (50, 51), and related reference genes retrieved from NCBI using BLAST (49) was assessed through multiple sequence alignment with MAFFT (v.7.525) (52), and the results were visualized as a phylogenetic tree constructed using FastTree (v.2.1.11) (53) (Supplemental Methods; Fig. S1).

### Methylation analysis for *sul4* contigs and wider set of contigs

Methylation analysis was run for all 12 *sul4* contigs sequenced and assembled here. More detailed methods for methylation analysis are found in the Supplemental Methods. Briefly, PacBio reads with kinetics tags were mapped against the assembled contigs, and the kinetics information in the mapping reads was summarized by ipdSummary (v.3.0) (https://github.com/PacificBiosciences/kineticsTools/). The MultiMotifMaker algorithm (54) was run to predict the sequence motifs surrounding the methylated bases. The methylation motifs of types m6A and m4C were explored and visualized using pheatmap (v.1.0.12) (55) in R (44) and RStudio (v.2024.12.0+467.pro1) (45).

To explore contigs with similar methylation profiles, methylation analysis was run for a wider set of contigs from all wastewater samples. However, due to the resource-intensive nature of *de novo* motif prediction, only a subset of the contigs from the entire assembly could be analyzed for methylation profiles (Fig. S3). During the exploration of methylation profiles similar to *sul4*-containing contigs, those matching only the GATC (m6A) motif were excluded. This approach was chosen because this motif is highly prevalent due to its regulatory roles across bacterial taxa (36), which could overshadow patterns of other motifs (36, 56–58). We tested the performance of MultiMotifMaker in *de novo* predicting methylation motifs in mixed communities with partially known bacterial composition by whole-genome sequenced community members (59, 60) (Supplemental Methods; Fig. S2).

### Gene annotations of *sul4* sequences

The bacterial genome annotation tool Bakta (v.1.7.0) (61) was run for all *sul4* sequences, including those sequenced here and those obtained from public sequence databases, to get the coordinates for *sul4* genes in these sequences. Sequence region 10 kbp up- and downstream of the *sul4* gene was extracted by a custom script applying SeqKit tools (v.2.5.1) (62). After dereplication of *sul4* sequences sharing above 90% similarity using CD-HIT (v.4.8.1) (63), Proovframe (v.0.9.8) (64), applying Diamond (v.2.0.15) (65), was used to identify and correct indels causing frameshifts in coding sequences and affecting gene prediction. Finally, the remaining 34 ~20 kbp *sul4*-flanking sequences were visualized using clinker (v.0.0.27) (66).

### Investigation of *sul4* module genes

Integron integrase genes from *sul4* sequences were extracted and aligned, along with five reference integrase genes from NCBI, using MAFFT (v.7.505) (52). Half of the *sul4* sequences (*n* = 17) were included in the integrase gene sequence similarity analysis, as in some cases, the contig or read was cut at the gene site. The reference genes were selected based on their similarity to integrase genes from different branches of the initial tree, identified through a BLAST search against the NCBI database. Additionally, a reference gene of class 1 integron integrase encoded by *Escherichia coli* EC261 (AP027420.1) was included in the analysis. The multiple sequence alignment was rooted with site-specific recombinase genes *xerC* (NC_000913.3:3996287–3997183, GeneID: 948355) and *xerD* (NC_000913.3:3038847–3039743, GeneID: 947362) in *Escherichia coli* str. K-12 substr. MG1655. Like integron integrases, these genes are tyrosine recombinases that mediate site-specific DNA recombination (67). The phylogenetic tree was drawn using RAxML (v.8.2.12) (68) with model GTRGAMMA and visualized with Interactive Tree of Life (iTOL) (69). The *sul4* module IS*CR* sequences were extracted through the detection of *ori*IS and *ter*IS sequences specific to these elements (9) (Supplemental Methods). HattCi (v.1.0b) (70) was used to investigate the *attC* sites as putative integron-associated target sequences for the IS*CR* elements. The relatedness of the IS*CR* sequences present in our *sul4* modules, compared to the known IS*CR* genes and canonical IS*91* family members (9), was studied using multiple sequence alignment with Clustal Omega (71). The analysis of the phage-related genes involving geNomad (72) and iPHoP (73) is found in the Supplemental Methods.

### Plasmid prediction

The plasmid contigs from wastewater metagenomes were predicted using two programs, plasX (74) and geNomad (v.1.8.0) (72), to determine whether the detected *sul4* genes were located on plasmids and to filter data for the broader methylation analysis, for which only putative plasmid contigs longer than 30 kb were considered (see “Methylation analysis for *sul4* contigs and wider set of contigs”). For plasX, Anvi’o (v.8) (75) was used to generate a contigs database with functional annotations from COG (database version COG_2014) and Pfam (database version Pfam_v32) databases before running the plasX predictions. The geNomad program was run with the default parameters.

### Curation of MAGs for *sul4* carriers

MAGs containing the *sul4* gene were curated through manual binning using Anvi'o (v8) (75) integrated into a custom Snakemake (48) workflow. The whole process was done separately for the two different assemblies generated by hifiasm-meta and metaFlye. A more detailed description of the programs and analysis scripts used is found in the Supplemental Methods. In brief, functional gene annotations, including ARG annotations with ResFinder (v.4.2.3) (76), were done for contigs databases built according to Anvi’o workflow. Illumina short reads were recruited to gather the reads aligning to the contigs. MAGs for *sul4*-carrying contigs were manually refined within the Anvi’o interactive interface, visualizing each metagenomic sample at a time. Quality check with CheckM2 (77), as well as taxonomical identification with GTDB-Tk (database version 220) (78), was done for the manually refined *sul4* MAGs. MAGs fulfilling the criteria of completion and redundancy values of 50 and 10, respectively, were kept for the preliminary analysis. Unique high- and middle-quality MAGs were described in more detail.

### Analysis of non-*sul4* contigs and MAGs

The non-*sul4* contigs with similar methylation profiles to some of the *sul4* contigs were analyzed similarly to the *sul4* contigs regarding plasmid prediction and the analysis of the *sul4*-flanking region. One of these five contigs, which showed a similar phage sequence region to some of the gene *sul4*-encoding contigs, was investigated in more detail and visualized alongside the *sul4* contigs. As these five contigs originated from two different samples (SLU1 and SLU2), they were combined sample-wise into two separate MAGs of two and three contigs. The resulting MAGs were assessed for quality using CheckM2 (77), and their taxonomic identity was determined with GTDB-Tk (78).

## RESULTS

*sul4* was detected in all but one of the wastewater samples, including influent, effluent, and dried sludge. Most of the metagenomic reads mapping to *sul4* originated from sludge samples, while reads from influent and effluent were fewer (Table 1). Moreover, *sul4*-containing contigs could be obtained only from sludge metagenomic assemblies (Table 2), where *sul4* was more commonly detected and the bacterial diversity was decreased in contrast to the influent (Fig. S4). The average length of *sul4* contigs ranged from 8,636 to 1,884,747 bp (Table 2). Four and seven contigs with *sul4* were constructed using the two different assemblers, hifiasm and metaFlye, respectively (Table 2). According to the plasmid prediction analysis with plasX and geNomad, only one of the *sul4* contigs (contig_s43139.ctg070500l) showed plasmid-like properties, and this was identified solely by geNomad (Table 2). However, this contig was notably shorter than the other *sul4* contigs (8,636 bp), which might have affected the plasmid prediction algorithms’ abilities to correctly identify plasmid sequences.

**TABLE 1:** Reads mapping to reference gene *sul4* (NG_056174.1)

Sample code	Sample	Reads mapping to *sul4*
INF1	Influent	1
INF2	Influent	0
INF3	Influent	3
EFF1	Effluent	3
EFF2	Effluent	1
EFF3	Effluent	1
SLU1	Sludge	23
SLU2	Sludge	34
SLU3	Sludge	8

**TABLE 2:** Assembly statistics of *sul4* contigs^*a*^

Sample	Assembler	No. of contigs	Contig names	Contig length (bp)	Score for plasmid plasX/geNomad
SLU1	hifiasm	1	contig_s1.ctg000044l	1,884,747	0/0
SLU2	hifiasm	1	contig_s3.ctg001711l	109,015	0/0
SLU3	hifiasm	2	contig_s3566.ctg005183l; contig_s43139.ctg070500l	314,103 8,636	0/0 0.003/0.97
SLU1	metaFlye	2	contig_13571; contig_73809	525,812 143,767	0/0 0/0
SLU2	metaFlye	4	contig_14681; contig_49547; contig_49550; contig_49551; contig_64719	50,817 19,369 38,474 396,357 793,955	0/0 0.007/0 0/0 0/0 0/0
SLU3	metaFlye	1	contig_65443	131,210	0/0

^*a*^ The plasX scores below 1 × 10^−3^ are denoted as 0.

### The conserved module containing *sul4*, truncated *fol*K, and an IS*CR* element was found in diverse genetic contexts

**Fig 1:**
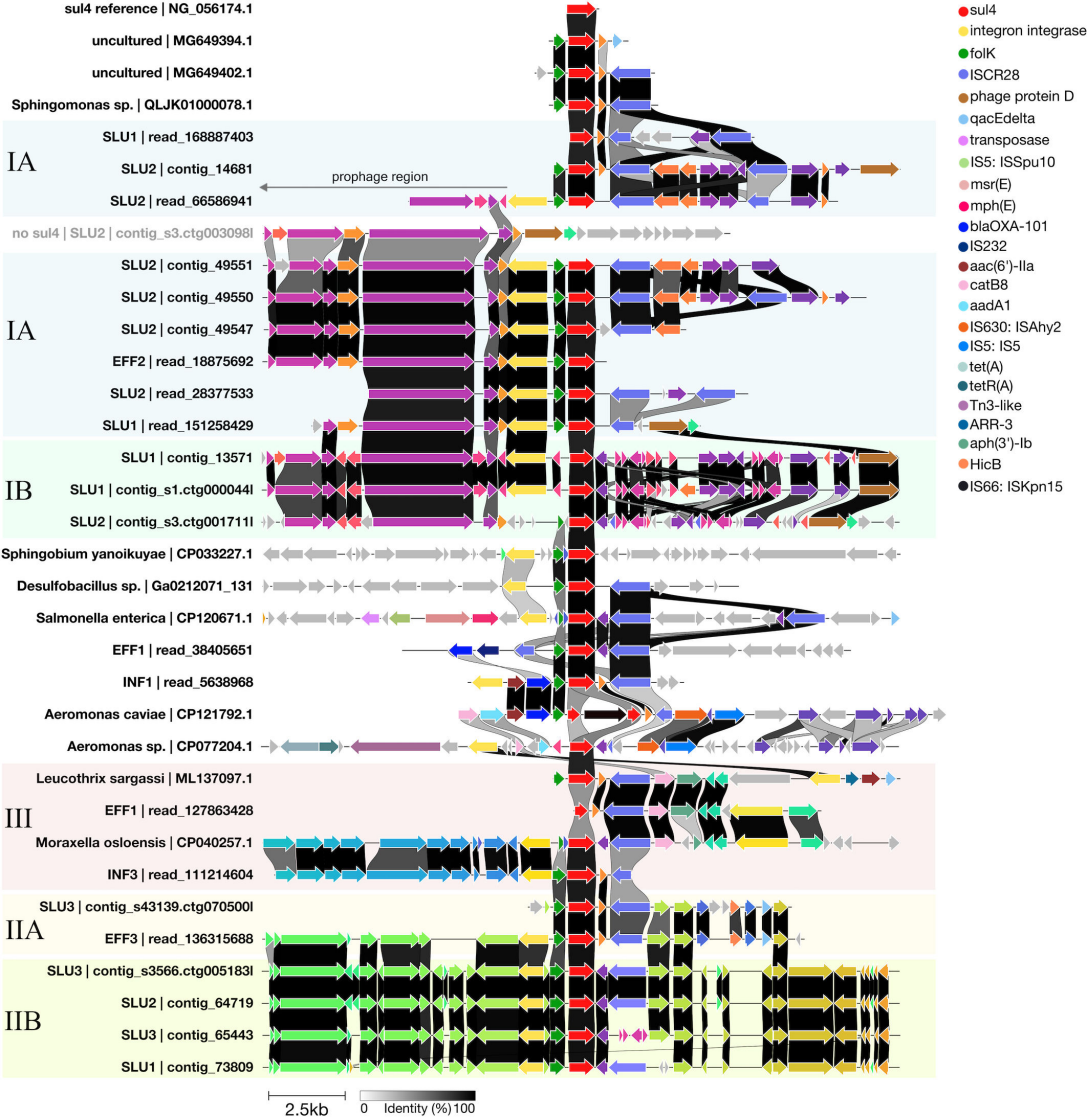
Genetic contexts of 10 kb upstream and downstream of the *sul4* gene in long reads and contigs sequenced here and *sul4* accessions published previously. Visualization was done using Clinker based on Bakta annotations. The alignments are centralized on the *sul4* gene, colored red. ARGs and key mobile genetic elements are color-coded and explained in the legend on the upper right. Other gene annotations without a specified color label are displayed using the default colors by Clinker, which assigns consistent colors to genes with similar sequences. Vertical links indicate the similarity of genes between different sequences (black = identical; white = no similarity).

A highly conserved gene region flanking the *sul4* (hereafter *sul4* module) was identified in all *sul4* reads and contigs from our wastewater samples. Furthermore, with a few exceptions where the sequence was cut, probably leaving out some of the genes, an integron integrase was always linked to the *sul4* module (Fig. 1). The core structure of the *sul4* module consisted of a partial *fol*K gene, *sul4,* and an IS*CR* element found downstream of *sul4* (Fig. 1). To further investigate the conservation, variation, and potential role of this module in mobilizing *sul4*, we expanded our analysis to include sequences encoding *sul4* found in public sequence databases. Screening sequence data in the NCBI and IMG with reference gene *sul4* (NG_056174.1) resulted in 11 different hits with a good match (length above 500 bp of the *sul4* gene length, 864 bp). These matches included the already published *sul4* sequences by Razavi and colleagues (NG_056174.1, MG649394.1, MG649402.1, uncultivated bacteria) (15) and Peng and colleagues (CP120671.1, *Salmonella enterica*) (23), as well as those sequences without previous description regarding their *sul4* carriage (Table 3). A highly conserved *sul4* module was present in all of these sequences. We also noted the reports of *sul4*-resembling genes published by Shindoh and colleagues (51) and Su and colleagues (50). However, we observed only low amino acid similarity between these 24 sequences and the reference gene *sul4* (NG_056174.1) (sequence similarity ranging from 34.45% to 47.64%) (Fig. S1). In fact, they showed greater similarity to *folP* genes (Fig. S1), the ancestral source of the *sul* genes (79). Hence, we concluded that these genes may possess functionality that is different from sulfonamide resistance. The absence of mobile genetic elements in the surrounding regions of these sequences further supported this hypothesis and led us to exclude the sequences from these two studies from our analysis.

**TABLE 3:** Reference sequences for data visualized in Fig. 1

Name	Source	Location	Year	Species	Study reference
NG_056174.1	River sediment	India, Patancheru, Hyderabad	2016	Uncultured	(15)
MG649394.1	River sediment	India, Patancheru, Hyderabad	2016	Uncultured	(15)
MG649402.1	River sediment	India, Pune, Maharashtra	2016	Uncultured	(15)
CP077204.1	NA	USA, Maryland	NA^*b*^	*Aeromonas* sp. FDAARGOS 1402, plasmid	Unpublished^*c*^
CP121792.1	*Homo sapiens*: plasma	Taiwan (hospital)	2020	*Aeromonas caviae* strain FIONA 16800, plasmid	Unpublished^*d*^
Ga0212071_131 (BioProject PRJNA471375)	Bioreactor: sludge microbial community	Hong Kong	2017	*Desulfobacillus sp008933825* (MAG)	(80)
ML137097.1 ^*a*^	Brown seaweed	China, Yantai, Yellow Sea	2017	*Leucothrix sargassi* C3212	(81)
CP040257.1 ^*a*^	Mycoplasma culture contaminant	NA	1990	*Moraxella osloensis* MOXF1	Unpublished^*e*^
CP120671.1	*Homo sapiens*: feces	China, Guangyuan	2020	*Salmonella enterica* SC2020597	(23)
QLJK01000078.1a	*Homo sapiens*: cerebral spinal fluid	USA	2016	*Sphingomonas* sp. S-NIH.Pt3_0716	(82)
CP033227.1	Contaminated soil	China, Shanghai	2017	*Sphingobium yanoikuyae* SJTF8, plasmid	(83)

^*a*^ Suppressed accessions in the RefSeq database due to putative contamination or unconfirmed source organism.

^*b*^ NA, not available.

^*c*^ Data submission by D. Rasko, L. Tallon, L. Sadzewicz, X. Zhao, J. Boylan, S. Ott, H. Bowen, K. Vavikolanu, A. Mehta, J. Aluvathingal, S. Nadendla, and Y. Yan.

^*d*^ Data submission by Y.-T. Huang.

^*e*^ Data submission by D. R. Brown, D. L. Michaels, G. F. Kutish and S. Frasca Jr.

The source environment of *sul4* sequences from databases varied greatly, ranging from anthropogenically contaminated river sediment to seashore marine seaweed, as well as clinical isolates of human origin and microbes from wastewater bioreactors (Table 3). These species included opportunistic human pathogens *Salmonella enterica*, *Aeromonas* spp., as well as *Sphingomonas* sp. and *Moraxella osloensis* (Table 3), which also contain pathogenic strains, though less frequently. The remaining *sul4* carrier bacteria from sequence databases included the species *Leucothrix sargassi*, *Sphingobium yanoikuyae*, and *Desulfobacillus sp008933825*. However, as an important note, three of the NCBI reference sequence (RefSeq) accessions for *sul4* carrier bacteria—GCF_005518075.1 for *Moraxella osloensis* MOXF1 (CP040257.1), GCF_004010795.1 for *Leucothrix sargassi* C3212 (ML137097.1), and GCF_003950715.1 for *Sphingomonas* sp. S-NIH.Pt3_0716 (QLJK01000078.1)—had been suppressed as they did not meet the criteria of RefSeq data. For instance, for the *M. osloensis* strain MOXF1, the assembly coverage was 70.84%, which indicates that ~30% of this genome is not *M. osloensis*. Instead, for *L. sargassi* C3212, ~30% of the genome is a good match for *Moraxella tetraodonis,* referring to contamination in this genome. Finally, the RefSeq accession for *Sphingomonas* sp. S-NIH.Pt3_0716 had been suppressed as the source organism could not be confirmed. This information was considered when evaluating the reliability of these three genomes. Geographically, the *sul4* sequences found in sequence databases originated from different parts of the world, with an emphasis on Asian countries (Table 3).

Wider genetic contexts surrounding *sul4*, including sequences 10 kb upstream and downstream of the *sul4* gene, were analyzed and visualized for all *sul4* sequences (Fig. 1; Table 3). The genetic contexts beyond the more conserved *sul4* modules exhibited greater variation and could be classified into three major groups (I–III) by visual inspection, each showing high similarity in gene content (Fig. 1). More specifically, groups I and II contained sequences with moderate similarity, which led to their division into subgroups IA-B and IIA-B. The remaining wider genetic contexts were either unique or too short to be described as members of any of the groups I–III (Fig. 1).

### Subtle sequence variations in *sul4* modules and adjacent integron integrases were observed across the different contexts

**Fig 2:**
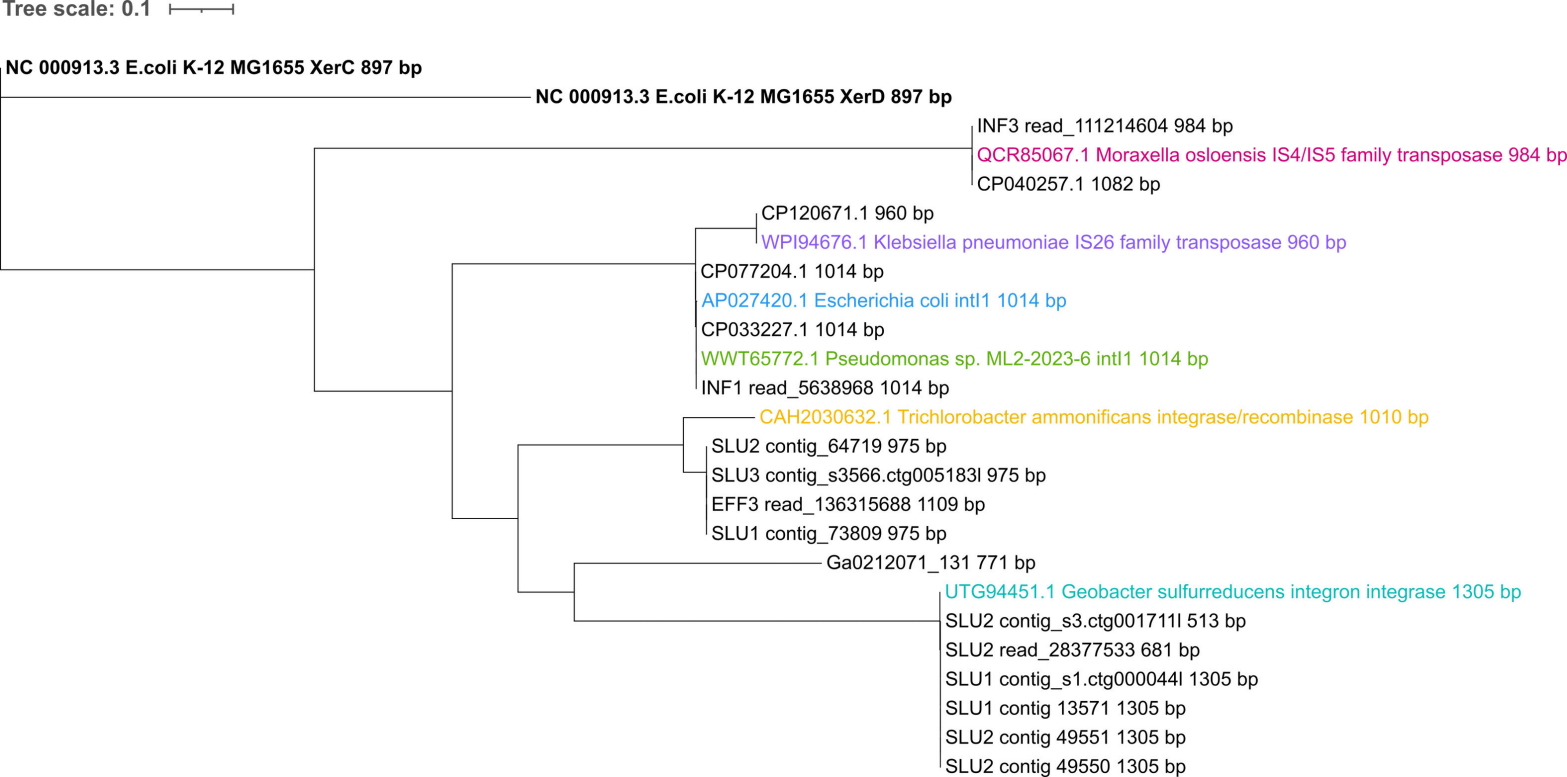
Phylogenetic tree of integron integrase sequences in *sul4* sequences. The tree was rooted with XerC and XerD recombinase-encoding genes in type strain *E. coli* K-12 MG1655 (bold). Multiple sequence alignment was run using MAFFT, and the tree was computed with RAxML. iTOL was used for visualization. The other reference integrase genes are highlighted with colors other than black.

Although the core structure of *sul4* was highly conserved among all investigated sequences, slight differences were also noted (Fig. 1). First, the comparison of the integrase sequences upstream of the *sul4* module resulted in roughly six clusters (Fig. 2). These clusters were accompanied by a reference integrase gene from previously published sequences (Fig. 2). An exception to this was the *Desulfobacillus sp008933825* (Ga0212071_131) encoding integrase gene, which was shorter and most different from any of the other integrases (Fig. 2). Integrase genes from *sul4* sequences, classified as group I based on their broader genetic context (Fig. 1), were grouped into a single cluster (Fig. 2). This cluster was represented by the reference integrase gene from *Geobacter sulfurreducens* (Fig. 2). Integrases of *sul4* sequences of group II (Fig. 1) showed the closest, but not perfect, similarity with the *Trichlorobacter ammonificans* integrase reference gene (Fig. 2). A third cluster was formed by integrases from one influent wastewater read and the *M. osloensis* (CP040257.1) strain, both belonging to group III (Fig. 1). This cluster was represented by a reference integrase gene from another *M. osloensis* strain (Fig. 2).

The separate cluster of the integrases in *Aeromonas* spp. (CP077204.1) and *Sphingobium yanoikuyae* (CP033227.1) was accompanied by one influent read and reference integrase genes of class 1 integron integrase (*intI1*) found in *Pseudomonas* sp. and *E. coli* (Fig. 2). According to a BLAST search against the NCBI database, identical *intI1* genes are also found in many other gram-negative opportunistic human pathogens of class Gammaproteobacteria (data not shown). The integrase gene in *sul4* host *S. enterica* (CP120671.1) showed high similarity with the reference integrase gene hosted by gram-negative opportunistic pathogens such as *Klebsiella pneumoniae*. These two integrases showed the highest similarity with the *intI1* cluster.

Additional ARG properties beyond *sul4* were detected in part of the sequences. Specific to the *Sphingobium yanoikuyae* strain (CP033227.1), a putative aminoglycoside riboswitch linked to the integron *attI* site was identified between the integrase and *sul4* (Fig. 1), while some of the ARGs *aac(6′)-IIa, bla*OXA-101*, catB8,* and *aadA1* were found to be part of the *sul4*-associated integron cassettes in one influent wastewater read and *Aeromonas* spp. plasmid sequences (Fig. 1). Specific to the group III sequences, also secondary integrases with ARG arrays that were located more distant downstream of *sul4* were observed (Fig. 1). Altogether, the variation observed in the arrangement and phylogeny of these integrase sequences, extending beyond those of class 1 integrons, suggests their enduring role in linking and potentially mobilizing *sul4* across different contexts.

According to our phylogenetic analysis, the IS*CR* elements present in nearly all *sul4* modules investigated here, across various wider contexts, were identified as IS*CR28* (9), as they were dissimilar to the other IS*CR* elements, including IS*CR20*, and canonical IS*91*-like genes (Fig. S5). We detected both *ori*IS and *ter*IS or at least *ori*IS sequences in all these IS*CR28* elements (data not shown). Also, integron-associated *attC* sites were identified at the end or right after each IS*CR28* gene (data not shown). As an important note, although in Fig. 1 the gene annotations for IS*CR28* in group IB, as well as some of those in group IIB, appear substantially distinct from the IS*CR28* in the other sequences, our phylogenetic analysis confirmed that they are identical, differing only slightly in length (Fig. S5 and Table S4). This incorrect visualization was caused by the frameshift changes affecting gene annotation. Secondary IS*CR* genes further on the right-hand side were common to many group I sequences and *S. enterica* (CP120671.1) (Fig. 1). Instead, in one effluent read, an additional remnant of an IS*CR28* element was located also upstream of *sul4* next to the *bla*OXA-101 gene (Fig. 1).

However, in three *sul4* modules, the IS element was dissimilar to IS*CR28* and IS*CR20*: These elements, found in the two plasmids of *Aeromonas* (CP121792.1 and CP077204.1), and *Sphingobium yanoikuyae* (CP033227.1) grouped into their separate clusters (Fig. S5). The BLAST search of the IS element of *S. yanoikuyae* (CP033227.1) revealed that part of this gene is found primarily in other members of *Sphingobium* and related genera (data not shown). The IS element genes in the *Aeromonas* spp. *sul4* plasmids were, in fact, annotated as three genes: IS*630*-like IS*Ahy2* transposase, hypothetical protein-encoding gene, and IS*5*-like IS*5* element. According to a BLAST search against the NCBI database, similar gene regions are found in various other Gammaproteobacteria, including opportunistic pathogenic genera such as *Pseudomonas, Salmonella, Klebsiella,* and *Enterobacter* (data not shown).

Taken together, IS*CR28* was found in most of the *sul4* modules investigated. However, two other kinds of clearly distinct IS sequences at this location were found: one in the *sul4*-carrying *Aeromonas* plasmids (and found in other typical opportunistic pathogen Gammaproteobacteria) and the other one hosted by a plasmid of *Sphingobium yanoikuyae* and found in closely related taxa. Compared to the integrases and IS elements discussed above, fewer dissimilarities were detected for the gene *sul4* itself (Fig. 1). However, one example was the *Aeromonas caviae* FIONA 16800 (CP121792.1), where the *sul4* gene was split into two parts by the incorporation of the IS*66*-like IS*Kpn15* transposase gene (Fig. 1).

### *sul4* modules were identified in various wider contexts, exhibiting minimal to moderate similarity to one another

Analysis with geNomad revealed that the *sul4* module in group I (both subgroups IA and IB) sequences, including reads and contigs from effluent and sludge samples, was located adjacent to a prophage sequence region (Tables S1 to S3). Part of this region is visible in Fig. 1, downstream of the integrase gene in group I sequences (genes on the left-hand side of group I in Fig. 1). These prophage sequences were identified to belong to class Caudoviricetes (Table S1).

The sequence regions examined in groups IIA and IIB were highly similar, with the main difference being the presence of type 2 toxin-antitoxin system HicA and HicB family toxin-encoding genes downstream of IS*CR28* in group IIA and their absence in group IIB (Fig. 1). Group III was formed of both sequences of this study and those from the public sequence databases (Fig. 1). The two reads from influent and effluent wastewater resembled the wider genetic context of *sul4* seen in *Moraxella osloensis* (CP040257.1) and *Leucothrix sargassi* C3212 (ML137097.1) (Fig. 1). More specifically, the influent read aligned with the upstream, and the read from effluent with the downstream sequence of *sul4*. The region further downstream of *sul4* contained another integron cassette with *ARR-3*, *aac(6′)-Ib*, *qacEdelta1,* and *sul2* (data not shown). However, in *L. sargassi*, a gene encoding a protein of unknown function seemed to truncate the secondary integron cassette (Fig. 1).

In addition to the groups described above, a miscellaneous set of genetic contexts, sharing varying levels of similarity among each other, was observed (Fig. 1). These sequences included the recently described, known *sul4* carrier *Salmonella enterica* (CP120671.1), and those found in public databases without previous reports on their *sul4* carriage: *Sphingobium yanoikuyae* (CP033227.1), *Sphingomonas* sp. (QLJK01000078.1), *Aeromonas* spp. plasmids of two different strains (CP077204.1 and CP121792.1), and *Desulfobacillus sp008933825* (Ga0212071_131). The two distinct *Aeromonas* strain plasmids carrying *sul4* showed similar genetic composition to each other and partially with one influent wastewater read. The remaining *sul4* sequences identified as *S. enterica*, *D. sp008933825, S. yanoikuyae*, and *Sphingomonas* sp. were all unique regarding the wider genetic context of *sul4* (Fig. 1).

### Unique methylation profiles indicated the presence of distinct *sul4* hosts in wastewater

**Fig 3:**
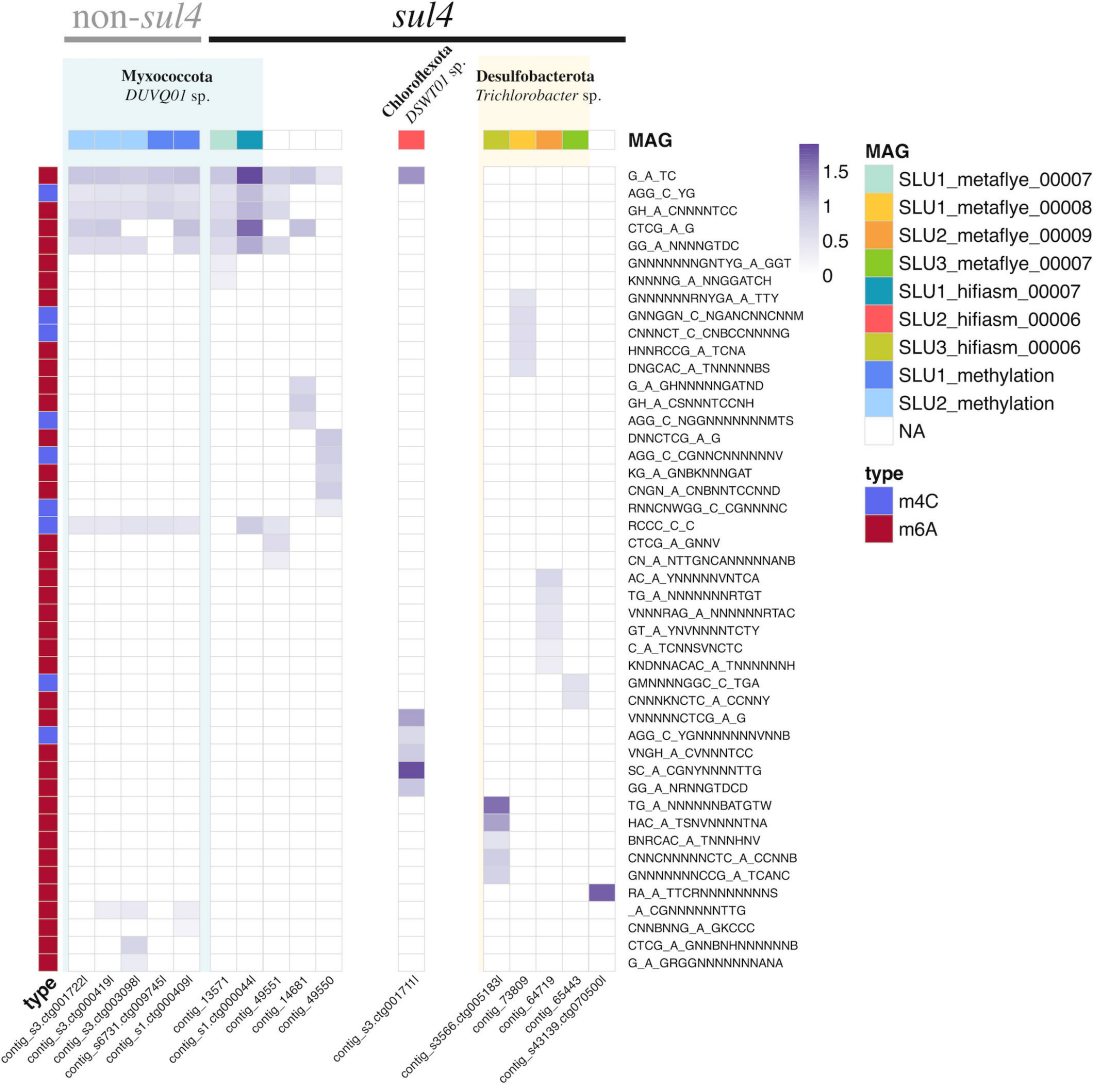
Methylation profiles of *sul4* contigs and contigs without *sul4*. Contigs are represented as columns, and the methylation motifs are shown as rows on the right-hand side of the heatmap. The intensity of the purple color in the heatmap shows the fraction of the methylated sites for each of the motifs predicted by the MultiMotifMaker algorithm (54). The modification type (m6A or m4C) is indicated by color coding on the left-hand side. The top-row annotation indicates the MAG in which each contig is included, if applicable, and the taxa assigned to the MAGs are shown in the text.

After finding the conserved *sul4* module in different wider contexts in our wastewater samples, we were interested in identifying the bacterial species carrying these sequences. For that, we exploited bacteria-specific methylation patterns of the *sul4*-encoding contigs. On average, six methylated motifs (ranging from 1 to 13) of types m6A and m4C were detected among the *sul4* contigs (Fig. 3). One or more methylated motifs were detected for all but one *sul4* contig (contig_49547), for which no methylated motifs were identified (Fig. 3). The most common methylated motif, detected in six *sul4* contigs, was GATC. A distinctive cluster was formed of *sul4* contigs possessing the methylated motif GATC together with at least one of the five other motifs, AGGCYG, GHACNNNNTCC, GGANNNNGTDC, CTCGAG, and RCCCCC (Fig. 3). The two other *sul4* contigs with the methylated motif GATC (Fig. 3) lacked any other motifs of this cluster. The remaining five *sul4* contigs had no methylated motifs in common with any of the other contigs (Fig. 3). The methylation-based grouping of contigs into three categories—those with a uniform profile based on several motifs, those with GATC but otherwise a unique profile, and those with no consistent pattern (Fig. 3)—suggested that at least two distinct bacterial species were carrying the *sul4* gene in our wastewater samples.

To test the discriminatory power of methylation signatures for binning species-specific contigs and to find contigs of common origin species with *sul4* contigs, a wider set of contigs was examined in search of similar methylated motifs displayed by the 12 *sul4* contigs. For that, methylation profiles of large chromosomal contigs above 200 kbp and putative plasmid contigs above 30 kbp in length of the hifiasm assembly were explored. Such stringent filtering was necessary due to the computationally intensive *de novo* methylation motif finder algorithm, which could only be run on a subset of the entire metagenomic data set. Applying these criteria resulted in a small subset of contigs from the full assemblies (1.38%) being retained for the methylation analysis (Fig. S3). The proportion of large contigs studied for methylation was higher in sludge samples, while the number of putative plasmid contigs was elevated in influent and effluent samples (Fig. S3). Using this approach, we identified five non-*sul4* contigs with methylation profiles matching those of the *sul4* cluster, all exhibiting virtually uniform profiles (Fig. 3). We therefore hypothesized that these contigs originated from genomes of the same bacterial species. To confirm this and further investigate the taxonomical identity of these sequences, we next focused on generating MAGs for these contigs.

### The presence of three distinct bacterial hosts for *sul4* in wastewater sludge was proven by metagenome-assembled genomes

MAGs were generated to verify the distinct *sul4* host taxa in wastewater that were indicated by the methylation profiles. Binning of *sul4* MAGs from three sludge samples and for assemblies from both assemblers was done using Anvi’o (84) (Table 4). Altogether, seven MAGs with *sul4* were generated. Two of them (SLU2_metaflye_00009 and SLU1_metaflye_00007) met the criteria for a high-quality MAG (>90% completeness, <5% redundancy) defined by the Genome Standards Consortium and verified by CheckM2 (Table 4). The remaining five MAGs were medium quality, with 50%–90% completeness and less than 10% contamination (Table 4). Both high-quality *sul4* MAGs originated from metaFlye assemblies of two sludge samples (SLU1 and SLU2) and represented genomes from two distinct bacterial phyla: Desulfobacterota and Myxococcota (Tables 4 and 5). More specifically, according to the taxonomic assignment by GTDB-Tk, the Desulfobacterota MAG matched with the reference genome GCF_933509905.1 of *Trichlorobacter ammonificans* belonging to the class Desulfuromonadia (Table 5). Similarly, the Myxococcota MAG matched with reference genome GCA_012840965.1, assigned to species *DUVQ01 sp012840965* of class UBA9042 (Table 5).

**TABLE 4:** Manual binning of *sul4* MAGs and Myxococcota MAGs without *sul4* generated based on contigs with shared methylation profiles^*a*^

MAG	C	R	Total length (bp)	No. of contigs	N50	GC content	*sul4* contig
¹Desulfobacterota							
SLU2_metaflye_00009	97.42	1.01	2,987,219	14	382,661	61.14	contig_64719
SLU3_metaflye_00007	82.91	2.64	2,803,798	53	78,501	60.97	contig_65443
SLU1_metaflye_00008	56.32	0.95	1,812,284	34	72,830	61.16	contig_73809
SLU3_hifiasm_00006	52.75	0.11	1,934,620	14	273,784	60.90	contig_s3566.ctg005183l
²Myxococcota							
SLU1_metaflye_00007	95.09	3.41	3,257, 935	14	355,039	50.77	contig_13571
SLU1_hifiasm_00007	89.18	1.53	3,016,815	7	1,884,747	50.83	contig_s1.ctg000044l
SLU2_methylation (contig_s3.ctg001722l; contig_s3.ctg000419l; contig_s3.ctg003098l)	85.99	2.38	3,029,587	3	1,179,051	51.0	–^*b*^
SLU1_methylation (contig_s1.ctg000409l; contig_s6731.ctg009745l)	26.97	0.11	916,485	2	712,371	51.0	–
³Chloroflexota							
SLU2_hifiasm_00006	70.84	5.81	2,612,149	33	181,217	59.65	contig_s3.ctg001711l

^*a*^ C and R refer to completeness and redundancy values estimated by CheckM2. Superscript numbers 1–3 denote taxonomic identities, which are detailed in Table 5.

^*b*^ A dash denotes absence of *sul4* gene contigs.

**TABLE 5:** Taxonomic identities of the MAGs listed in Table 4 (denoted by numbers 1*–*3), as assigned by GTDB-Tk

Phylum	Class	Order	Family	Genus	Species	Closest reference accession
¹Desulfobacterota	Desulfuromonadia	Geobacterales	Pseudopelobacteraceae	*Trichlorobacter*	*Trichlorobacter ammonificans*	GCF_933509905.1
²Myxococcota	UBA9042	UBA9042	*DTJE01*	*DTJE01*	*DUVQ01 sp012840965*	GCA_012840965.1
³Chloroflexota	Anaerolineae	B4-G1	*SLSP01*	*DSWT01*		GCA_019873725.1

All except one (SLU2_hifiasm_00006) of the remaining medium-quality *sul4* MAGs matched these two reference genomes and were found in two sludge samples (SLU1 and SLU3) (Table 4). Lastly, the medium-quality MAG SLU2_hifiasm_00006, distinct from all other MAGs, exhibited the closest match to a genome from the *DSWT01* genus of the Chloroflexota phylum (Tables 4 and 5).

### Methylation-based binning revealed Myxococcota *DUVQ01 sp012840965* genomes lacking *sul4* gene

To confirm the identity of the non-*sul4*-containing contigs exhibiting similar methylation patterns as the *sul4*-containing cluster contigs in Fig. 3, these contigs were binned together according to their origin sample (SLU1 or SLU2), resulting in MAGs SLU2_methylation and SLU1_methylation (Table 4). Similarly to *sul4* MAGs SLU1_metaflye_00007 and SLU1_hifiasm_00007, these MAGs were assigned as Myxococcota species *DUVQ01 sp012840965* (Tables 4 and 5). While the SLU2_methylation MAG was of medium quality, showing 86.0% completeness and 2.4% redundancy values, SLU1_methylation was less complete (27.0% completeness and 0.11% redundancy) due to its short length. Hence, this bin was left out of further analyses (Table 4). Altogether, the taxonomical identity of all Myxococcota MAGs generated for both *sul4* and non-*sul4* was in line with the initial methylation-based grouping of these contigs (Table 5; Fig. 3). This allowed us to investigate the differences seen in the genomes of these species with and without *sul4* in more detail (Fig. 1). Interestingly, phage sequence regions similar to those contigs belonging to Myxococcota MAGs with *sul4* were observed also in those not carrying *sul4* (Fig. 1).

## DISCUSSION

In this study, we described several novel genetic contexts and host species for the recently discovered sulfonamide resistance gene variant *sul4* by the application of long-read metagenomic sequencing and unique methylation signatures. The three *sul4* hosts detected in wastewater samples were *DUVQ01 sp012840965* of the Myxococcota phylum*, Trichlorobacter ammonificans* of the Desulfobacterota phylum, and species of the genus *DSWT01* of Chloroflexota phylum. Moreover, by exploring data available in public databases, we identified additional *sul4*-carrying bacteria from various ecological niches and taxonomic lineages, such as genera *Aeromonas, Moraxella, Sphingobium,* and *Desulfobacillus*, on top of the previously described *sul4*-carrying *Salmonella enterica* (23).

The core structure of the sequence region flanking the *sul4* gene (*sul4* module) was highly conserved among different *sul4* hosts examined here and those described by previous research (15, 23), suggesting this module’s importance in the dissemination of *sul4* across bacterial lineages. The detection of the integron integrase gene in all studied *sul4* contexts (those where the sequence was intact at this site) increased the evidence for its speculated roles in the *sul4* gene expression regulation and mobilization (15, 23). Previous literature on integron-mediated spread of ARGs, including *sul4*, has focused on class 1 integrons (15, 85) due to their substantial role in accelerating the antibiotic resistance crisis (7), as they can carry resistance gene cassettes among clinically important bacteria and between these and environmentally sourced species. As a putative example of the latter case, we found *sul4* in association with *intI1* in an environmental species, *Sphingobium yanoikuyae,* in a similar manner to *Pseudomonas* sp., *Aeromonas* sp., and other gram-negative opportunistic pathogens. This could support the hypothesis of class 1 integron-mediated movement of ARGs between environmental and clinically significant bacteria. As *intI1*-linked *sul4* modules were present also in our wastewater data, within a genetic context similar to those in *Aeromonas* spp., this seems to represent a somewhat common gene arrangement.

However, according to our results, other less-studied types of integrons, typically originating from environmental settings, can also act as key contributors in *sul4* dissemination. Our analysis revealed four other than class 1 integron-associated types of integrase genes linked to the *sul4* module. These integrase lineages mostly aligned with their broader context sequences and host bacteria, suggesting species-specific adaptations. One of these was the integrase of our *DUVQ01 sp012840965* Myxococcota MAGs. Noteworthy, the closest reference gene matching this integrase was hosted by *Geobacter* species (phylum Desulfobacterota) and not by species of the Myxococcota phylum, as anticipated. This was likely due to the unexplored diversity of the newly established phylum Myxococcota, resulting in limited reference sequence databases that lack prior descriptions of Myxococcota-specific integrases.

Despite the strong co-occurrence of the *sul4* module with integron integrases, these elements are not mobile themselves (7, 85) and thus do not fully explain how *sul4* is translocated into new contexts and host bacteria. Instead, the IS*CR* element within the *sul4* module holds a prominent potential for this task (15, 23). In most *sul4* modules investigated here, the detected IS*CR* element represented one of the most recently described members of this group, IS*CR28* (9). Due to the *ori*IS and *ter*IS sequences and the rolling-circle mechanism they enable (8), these elements can translocate themselves and, in some cases, their adjacent sequences into new contexts (9). Interestingly, Yuan and colleagues described a diverse range of genetic contexts for those IS*CR28* elements where intact *ori*IS and *ter*IS sequences were present, while with an incomplete *ori*IS, the wider contexts of IS*CR28* were highly conserved (9). The authors deduced that, in the latter cases, IS*CR28* was not mobile but rather a passive element, co-transmitted through the activities of other MGEs (9). With this background, the intact *ori*IS sites in all IS*CR28* in our *sul4* modules, together with the fact that they were found in a wide variety of genetic backgrounds and host bacteria, would support its mobile nature in this context. Conversely, finding *attC* sites at or right after in most IS*CR28* would imply that these elements are passively shuffled as components of the integron cassettes adjacent to *sul4* modules. If IS*CR28* were mobile itself, the question remains as to whether it also mobilizes adjacent genes, namely *sul4*. In our data, the arrangement of IS*CR28* and *sul4* in one effluent read resembled the sandwich-like structure reported previously, enabling the translocation of another ARG (9). In this structure, a remnant IS*CR28* with additional *ori*IS also exceeds upstream *sul4,* and the fusion by the two sequential *ori*IS leads to the capture of the sandwiched ARG—in our case, *sul4* (9).

Finding other than IS*CR* elements, namely members of various IS families, in three different *sul4* modules suggests that a broader range of IS elements than previously thought could facilitate the transfer of *sul4* into new hosts, which is worrying given the substantial abundance and diversity of IS elements found in bacterial genomes (86). However, as all these three sequences with non-IS*CR28* IS elements were found in *sul4*-hosting plasmids, it may be that the IS elements are passively co-transmitted throughout the plasmid-mediated spread of *sul4*. Although we found evidence supporting the role of both integrase and IS*CR28* for the *sul4* module mobilization, there is still no definite conclusion for this mechanism.

Besides the presence of integrases and IS*CR28* flanking *sul4* and their absence in the same species MAGs lacking *sul4*, the most compelling evidence for the mobility of the *sul4* module lies in its presence across various contexts, including plasmids, chromosomal regions, diverse genetic backgrounds, and even among unrelated species. Two of the newly discovered *sul4* hosts found in this study*—DUVQ01 sp012840965* and *Trichlorobacter ammonificans*—represented the phyla Myxococcota and Desulfobacterota. These phyla were only recently established during the reclassification of former members of the diverse and non-monophyletic Deltaproteobacteria class, previously placed under the Proteobacteria phylum (87, 88). As the cultivation procedures of these species are not straightforward (89, 90), the advancements in culture-independent omics methods have increased our understanding of species diversity in these groups, as shown by the high number of uncultivated bacteria in both phyla (88, 91). Notably, in the NCBI database, the taxonomy for accession GCA_012840965.1, which is the reference genome of Myxococcota *DUVQ01 sp012840965* assigned by GTDB-Tk, is classified under *Oligoflexus* sp. of the order Oligoflexales. This nomenclature reflects the old and recently revised classification scheme for members of the class Deltaproteobacteria (87, 88).

Desulfobacterota and Myxococcota are widespread in microbial communities of soils and various aquatic environments, such as marine and freshwater habitats as well as wastewater (88, 92), and they are known for their diverse metabolic abilities and involvement in various nutrient cycles (87, 88, 92, 93). For instance, the unusual roles of *Trichlorobacter ammonificans* G1 in nitrate reduction processes were recently described within an activated sludge inoculum (93). In addition to the versatile secondary metabolite production (91), Myxococcota are most recognized for their active predatory members, whose functions within activated sludge communities in wastewater have been extensively studied (94). In contrast to protist predators, Myxococcota are more prey-selective, favoring, for instance, *E. coli* and *Pseudomonas putida* (94) and other gram-negative bacteria (95). This could play a considerable role in the dissemination of resistance genes such as *sul4* within a microbial community, as the proximity between bacterial cells facilitates horizontal gene transfer (96).

Similarly to Desulfobacterota and Myxococcota, the members of the Chloroflexota phylum, represented by the third *sul4* carrier bacterium found in our wastewater, are frequently encountered in diverse aquatic environments, including marine ecosystems, as well as in wastewater, particularly activated sludge (92, 97, 98). Diverse biochemical cycles have been described also among these bacteria (99). Intriguingly, based on the phylogenetic analysis of Sul4 and other similar DHP synthase protein sequences across bacterial lineages, in the context of the first discovery of *sul4*, Razavi and colleagues proposed further investigations for studying Chloroflexota as the putative original host of *sul4* (15), which supports our findings of the Chloroflexota bacterium as one of the *sul4* carriers also in our samples.

None of the other *sul4*-flanking regions examined in this study displayed similar genetic contexts to those found in *Trichlorobacter ammonificans* (Desulfobacterota), *DUVQ01 sp012840965* (Myxococcota), or *DSWT01* sp. (Chloroflexota) MAGs. Thus, the observation of phage sequences belonging to the ubiquitous (100, 101), ARG transmission-associated (10) class Caudoviricetes near the *sul4* module in both Myxococcota and Chloroflexota *sul4* MAGs raised the question about the phage’s putative role in mobilizing *sul4* across bacterial lineages. However, the comparison of two *DUVQ01 sp012840965* MAGs, with and without the *sul4* module, revealed that a somewhat similar phage region was observed in both, suggesting that these sequences are unrelated to the presence and mobilization of *sul4*. It may also be that the region rich in phage sequences serves as a favorable target for the incorporation of other transconjugant genetic material (102), such as via integrases and IS*CR28*. Likewise, the prophage sequence integration could occur at sites high in MGEs, explaining the similar structures observed in different genomes.

The *sul4-*encoding MAGs of all three species, *DUVQ01 sp012840965* (Myxococcota)*, Trichlorobacter ammonificans* (Desulfobacterota), and *DSWT01* sp. (Chloroflexota), were found in samples from dried sludge. However, individual reads with *sul4* were also detected in the influent and effluent. These findings suggest that bacteria carrying *sul4* entered the wastewater treatment system via influent and potentially enriched in the activated sludge due to changes in environmental conditions modulating the bacterial composition and diversity (30, 103). Moreover, the treatment conditions may have facilitated the transmission of *sul4* genes between different bacterial carriers. Finally, some of these genes or their carrier bacteria persisted throughout the treatment process, as they were still detectable in the effluent and dried sludge. The elevated genus-level bacterial diversity in the influent compared to dried sludge likely hindered the detection, or at least the metagenomic assembly, of *sul4* contigs in the influent.

In addition to species of Myxococcota, Desulfobacterota, and Chloroflexota, other common wastewater species, such as *Sphingobium yanoikuyae* and *Desulfobacillus sp008933825*, were among the *sul4* hosts based on our analysis of sequences from public databases. Similarly to the PAH-degradation-encoding genes found in the plasmid of *S. yanoikuyae* (83), the *sul4* gene within this plasmid could facilitate its rapid adaptation to new and diverse environmental stresses. Moreover, the putative aminoglycoside riboswitch identified within this *sul4* module refers to the regulation of ARG expression in response to aminoglycoside antibiotics and putative integration of further integron-encoded gene cassettes (104). However, no additional ARGs were yet detected at this site.

Species within the *Desulfobacillus* genus, such as *D. denitrificans*, are known for their role in anammox consortia, where they contribute to nitrogen gas production through the anaerobic oxidation of ammonium in wastewater sludge (22). The adaptation mechanism of this consortia to antibiotic stress was recently described (22). Along with the subsequent recovery in nitrogen removal during long-term sulfamethoxazole exposure, a notable increase in the detection of *sul* genes was observed in time, and the greatest increase was seen for *sul4* (22). As the authors speculate, the increased production of modified DHP synthase encoded by the *sul* genes would unlock the folate pathway, inhibited by sulfamethoxazole (22). Moreover, the interspecies cooperation seemed to play a crucial role in this mechanism, as evidenced by the increased frequency of MGE-mediated ARGs and the high genome plasticity enabling the transfer of adaptation-improving genes such as *sul* genes between the anammox consortia members and other sludge bacteria (22), which would further widen the host range of these genes in wastewater.

Conclusively, given the long half-life and low removal efficiency of sulfamethoxazole in traditional wastewater treatment processes (105), the presence of antibiotic residues could provide favorable conditions for accumulation, spread across environmental and clinically relevant bacteria, or even the emergence of novel *sul* variants. Depending on the intended use and discharge destination of effluent and dried sludge, the *sul4* genes and *sul4*-carrying bacteria found in them may spread further in the receiving microbial communities, increasing the environmental burden of human-induced resistance. Moreover, high concentrations of sulfonamide antibiotic residues have been measured in natural waters, especially in low- and middle-income countries (15, 106, 107), where the use of sulfonamides is pronounced (20) and the treatment of wastewater is less regulated or efficient (108). These conditions, resembling those in the original detection of the *sul4* gene in India (15), could give rise to the further emergence and spread of *sul* variants among environmental bacteria and potentially species relevant to human health.

Besides taxa related to wastewater, we identified several other *sul4* carriers among opportunistic human pathogens. To our best knowledge, the patient isolate *S. enterica* subsp. *enterica* from China in 2020 is so far the only bacterial isolate for which the phenotypic resistance of *sul4* has been demonstrated through laboratory studies using cloning vector pUC19 (23). This multidrug-resistant strain belonged to *S. enterica* serovar Thompson, a common cause of human infections and foodborne outbreaks (109). Curiously, Peng and colleagues demonstrated that the *sul4* gene in this strain was integrated into the chromosome from a hybrid plasmid consisting of type IncHI2/HI2A, common in gram-negative pathogens, and gene arrays rich in ARGs and MGEs (23). The evidence of *sul4* circulating in plasmids of common pathogenic species is concerning, as any new modes of dissemination for *sul4* will enhance the rapid spread of this gene. In addition to the prior plasmid phase of the *sul4* module in *S. enterica* and plasmid-encoded *sul4* in *S. yanoikuyae* (83), we identified two other distinct clinical *Aeromonas* spp. strains’ plasmids carrying *sul4*. Moreover, the additional ARGs within these *sul4-*containing integron cassettes could facilitate the co-selection, resulting in the simultaneous mobilization of multiple ARGs (4). Since at least one metagenomic read from our influent wastewater data set matched the genetic context of the *Aeromonas* plasmids, we speculate that this *sul4* carrier was also present in our samples, although we did not manage to assemble these reads into contigs or MAGs to support this conclusion.

Among the remaining wider contexts of *sul4*, we found matching genome sequences classified as *Moraxella osloensis* and *Leucothrix sargassi* strains (81) in both influent and effluent wastewater reads. However, some uncertainty remains about the taxonomic identity of these strains. The contamination in the latter reference genome suggested that, in fact, both *sul4* hosts belonged to the *Moraxella* genus. Unfortunately, we were unable to assemble any contigs to construct MAGs from the matching wastewater reads in our data set, to confirm the species identity of these *sul4*-carrying bacteria. However, despite the lack of taxonomical confirmation, finding these highly similar sequences in different data sets and geographical locations supports the widespread prevalence of this *sul4* module context.

In summary, we show that the recently recognized sulfonamide resistance gene, *sul4,* is carried by diverse bacteria representing various ecological niches and taxonomical lineages. The highly conserved *sul4* module, containing *fol*K, *sul4,* and an IS*CR28,* was linked to different integron integrase genes in all investigated sequences, suggesting that the module is involved in the mobilization of *sul4*; however, the exact mechanism remains to be fully confirmed. Beyond these elements, the possibility of additional mobility-inducing mechanisms, such as the transfer of plasmids, co-selection through integron cassettes with multiple ARGs, or potential phage-mediated transmission, raises further concerns regarding the effectiveness of crucially important sulfonamide antibiotics in human and animal medicine. Species involved in the essential biochemical cycles for nutrient removal in activated sludge, such as Myxococcota, Desulfobacterota, *Desulfobacillus*, and *Sphingobium,* were found to be significant contributors to the carriage of *sul4*. Therefore, the role of activated sludge in possibly enriching the host bacteria of sulfonamide resistance should be investigated in more detail. As many of these bacteria are also considered environmental taxa, our results provide further evidence for the ongoing discussion about the role of environmental bacteria in the currently seen emergence and spread of ARGs, for instance, occurring at wastewater treatment plants, where bacteria from different sources are mixed (13, 24, 110). Moreover, this study demonstrates that the *sul4*-carrying bacteria or their genes can pass through wastewater treatment and end up in treatment outlets, highlighting the need for ARG monitoring of WWTP discharges.

## Data Availability

The sequence data are available under accession number PRJEB83306 in the European Nucleotide Archive (ENA). The MAG sequences are available in Figshare at https://figshare.com/projects/Host_range_and_genetic_context_of_sulfonamide_resistance_gene_sul4/231059. The analysis scripts are shared in the publicly available GitHub repository at https://github.com/melinamarkkanen/sul4_project.

## AFTER EPUB

*[This article was published on 19 November 2025 with an error in the second supplemental file. The supplemental file was corrected in the current version, posted on 2 December 2025.]*

## SUPPLEMENTAL MATERIAL

Supplemental Methods spectrum.00857-25-s0001.docx Detailed study methods.

Supplemental Material spectrum.00857-25-s0002.docx Supplemental results and Figures S1 to S5.

Supplemental Tables spectrum.00857-25-s0003.xlsx Tables S1 to S4.

ASM does not own the copyrights to Supplemental Material that may be linked to, or accessed through, an article. The authors have granted ASM a non-exclusive, world-wide license to publish the Supplemental Material files. Please contact the corresponding author directly for reuse.
