# Supplementary material for: Sulfonamide resistance gene sul4 is hosted by common wastewater sludge bacteria and found in various newly described contexts and hosts
Source: Microbiol Spectr. 2025 Nov 19;14(1):e00857-25. doi: 10.1128/spectrum.00857-25 (PMC12772301; doi:10.1128/spectrum.00857-25)
Supplement: Supplemental Methods — Detailed study methods. [file spectrum.00857-25-s0001.docx]

**Supplementary Methods**

Sulfonamide resistance gene *sul4* is hosted by common wastewater sludge bacteria and found in various newly described contexts and hosts

Melina Markkanen^1,2^, Denise Pezzutto^1^, Marko Virta^1,2^, Antti Karkman^1,2^

^1^ Department of Microbiology, University of Helsinki, Helsinki, Finland

^2^ Multidisciplinary Center of Excellence in Antimicrobial Resistance Research, University of Helsinki, Helsinki, Finland

Contents:

Preliminary read processing

Sequence comparison of *sul* genes and *sul4* resembling genes

Analysis of bacterial diversity

Methylation analysis for *sul4* contigs and wider set of contigs

Gene annotations of *sul4* sequences

Phylogenetic analysis of ISCR element sequences

Prophage prediction

Plasmid prediction

Curation of metagenome-assembled genomes (MAG) for *sul4* carriers

References

Preliminary read processing

The two separate sets of HiFi reads were produced from subreads using PacBio BAM toolkit programs (<https://github.com/PacificBiosciences/pbtk>) such as ccs (v6.4.0) and bam2fastq (v1.0.0) and lima (v2.6.0) for demultiplexing (<https://github.com/PacificBiosciences/barcoding>) concatenated within two separate in-house Snakemake workflows (v7.17.1) (1). For those with kinetics tags, program ccs_kinetics_bystrandify (v1.0.0) (<https://github.com/PacificBiosciences/pbtk>) was run before the demultiplexing step with lima.

Sequence comparison of *sul* genes and *sul4* resembling genes

The 23 sequences (Table 2 in Shindoh et al. 2023 (2)) reported by Shindoh et al. 2023 (2) and one sequence (NDKM-034-1) reported by Su et al. 2023 (3) were queried with blastx (4) against the amino acid sequence of gene *sul4* (NG_056174.1) to investigate their sequence similarity to *sul4*. Additionally, these sequences and similar reference sequences obtained from NCBI were used to build a phylogenetic tree. For that, sequence alignment was done by MAFFT (v7.525) (5) and the tree was drawn using FastTree (v2.1.11) (6) (Supplementary Figure S1). *Escherichia coli* K12 *fol*P gene (NC_000913.3) was used to root the tree.

Analysis of bacterial diversity

Read-based taxonomical profiling for wastewater metagenomes was done using Metaphlan4 (v 4.1.1) (7) using default parameters with Illumina short-reads The result tables were merged and further processed with phyloseq (8) by transform_sample_counts function in RStudio (v4.1.1) (9). Shannon diversity indices were calculated for different taxa levels using vegan package (v 2.6-6.1) (10) and visualized using ggplot2 (v3.5.1) (11).

Methylation analysis for *sul4* contigs and wider set of contigs

The contigs generated by one assembler, hifiasm-meta fulfilling the following criteria were included in the methylation analysis: contigs with length above 200 kb or length above 30 kb and potential indications of being plasmid contigs. The methodology for screening the putative plasmid contigs is explained in the later section of the Material and Methods under the title ‘Plasmid prediction’. In brief, for the methylation analysis, PacBio long reads with kinetics tags, prepared as described earlier, were first aligned to the complete assemblies. The contigs of interest (meeting the criteria detailed above) and the reads mapping to them were extracted into individual files to be used as input files for ipdSummary (v3.0) (<https://github.com/PacificBiosciences/kineticsTools/>) summarizing the base modifications detected in the mapped reads. Finally, *de novo* detection of sequence motifs surrounding each methylated base was performed using MultiMotifMaker algorithm (12). The methylation motifs of unknown type referred to as ‘modified_base’ by ipdSummary were left out of the analysis due to the weaker distinctive signal coming from them in comparison to m6A and m4C to which we focused instead.

We tested our approach using a synthetic community with known bacterial composition (13). After metagenomic assembly of the community, methylation motifs of the assembled contigs were predicted similarly as described above, and contigs were ordinated based on their methylation profiles using t-distributed Stochastic Neighbor Embedding (t-SNE) (Supplementary Figure S2). Supplementary Figures S2A-E denote different community samples with different treatments. The a priori known taxonomic identities of the metagenomic contigs were color-coded in the ordination plot to assess whether contigs clustered correctly by species based on their methylation profiles (Supplementary Figure S2).

Gene annotations of *sul4* sequences

The bacterial genome annotation tool Bakta (v1.7.0) (14) was run for all *sul4* sequences including those sequenced here and those obtained from public sequence databases to get the coordinates for *sul4* genes in these sequences. Based on the start and end coordinates of the *sul4* gene, 10 kbp up and downstream *sul4* gene were extracted by custom script applying SeqKit tools (v2.5.1) (15). Next, the *sul4* reads and contigs originating from our wastewater samples were dereplicated to remove highly similar or identical sequences using CD-HIT (v4.8.1) (16) with a threshold of 0.90. Furthermore, to identify and correct indels causing frameshifts in coding sequences and affecting gene prediction Proovframe (v0.9.8) (17) applying Diamond (v2.0.15) (18) was run for *sul4* reads and contigs sequenced here. Gene annotations with Bakta were run again for the extracted, dereplicated, and frameshift corrected ~20 kbp *sul4* flanking regions, and these sequences were visualized using clinker (v0.0.27) (19). Six more sequences were excluded from the final visualization of *sul4* flanking regions manually based on their high similarity to other sequences. After the above-described steps, 34 sequences were left for visualization.

Phylogenetic analysis of ISCR element sequences

The ISCR sequences were extracted from the *sul4* flanking sequences by the identification of *ori*IS (5’-ACTGATAGGAACTGTCATTTC-3) and *ter*IS (GAACGGGCTTGTCCCGTGA-3') site sequences charachteristic for IS91 family insertion sequences including ISCR elements (20). These sequences were compared to other known ISCR element genes by multiple sequence alignment using Clustal Omega (21). The reference sequences used in the alignment are listed in Table S4.

Prophage prediction

The prophage sequence regions were identified with geNomad (v1.8.0) (22). For sequences where a prophage region was identified near the sul4 region, the presence of phage-related genes was manually investigated by browsing Bakta annotations for gene names containing "phage" or "capsid" within the -10 kbp to +10 kbp region flanking *sul4*. The examined phage-related genes included for instance phage protein D (UniRef50: A0A5K7Z9M7), phage tail protein (UniRef50: A0LPZ2), and phage major capsid protein (UniRef50: E1QHY6) (Table S3). Phage host prediction tool iPHoP (v1.3.3) (23) was used to study the host preferences of the detected phages.

Plasmid prediction

The plasmid contigs from wastewater metagenomes were predicted using two programs plasX (24) and geNomad (v1.8.0) (22) to determine whether the detected *sul4* genes were located on plasmids and to filter data for the broader methylation analysis, for which only putative plasmid contigs longer than 30 kb were considered (See section ‘Methylation analysis’). For plasX, first, a contigs database of all contigs of interest was built by applying Anvi’o (v8) (25) programs anvi-script-reformat-fast and anvi-gen-contigs-database. Next, functional annotations with programs anvi-run-ncbi-cogs (database version COG_2014) and anvi-run-pfams (database version Pfam_v32) were run for the contigs databases and exported by anvi-export-functions and anvi-export-gene-calls. The resulting tables were used as input files for plasX programs plasx search_de_novo_families and plasx predict. Ultimately, this resulted in plasmid scores indicating the likelihood that a given contig is a plasmid. The geNomad program was run with the default parameters for the *sul4* contigs for additional confirmation regarding whether these contigs were plasmid-associated.

Curation of metagenome-assembled genomes (MAG) for *sul4* carriers

MAGs containing gene *sul4* were curated through manual binning using Anvi'o (v8) (25) programs integrated into a custom Snakemake workflow (1). The whole process was done separately for the two different assemblies generated by hifiasm-meta and metaFlye. The main steps included in the workflow were generating the contigs database (anvi-gen-contigs-database) and creating a set of functional annotations to be imported to the contigs database; COGs (anvi-run-ncbi-cogs, database version COG_2014) and HMM (anvi-run-hmms) functional gene predictions as well as analysis of single-copy core genes and their association to taxonomy (anvi-run-scg-taxonomy). Furthermore, the gene calls (anvi-get-sequences-for-gene-calls) was annotated with antibiotic resistance genes found in ResFinder database (v4.2.3) (26) using BLAST (4) with an E-value of 1e-20 and imported to the contigs database to enable the detection of *sul4* gene encoding splits for downstream analysis. Illumina sort-reads were used for mapping the reads against the metagenomic assemblies by bowtie2 (v2.4.4) (27). Sorting and indexing the mapped reads were achieved by Anvi’o script anvi-init-bam. The read recruitment results among other measures such as average coverage and detection by contig, were stored into profile databases by anvi-profiling command, and the sample profiles were merged using anvi-merge. Next, MAGs for *sul4* carrying contigs were manually refined within the Anvi’o interactive interface visualizing each metagenomic sample at a time. The refined MAGs were summarized with anvi-summarize command. Quality check with CheckM2 (28) as well as taxonomical identification with GTDB-Tk (database version 220) (29) was done for the manually binned *sul4* MAGs. For those bins fulfilling the criteria of completion and redundancy values of 50 and 10 respectively were kept for the preliminary analysis. Unique high and middle-quality MAGs were described in more detail.

References:

1. Mölder F, Jablonski KP, Letcher B, Hall MB, Tomkins-Tinch CH, Sochat V, Forster J, Lee S, Twardziok SO, Kanitz A, Wilm A, Holtgrewe M, Rahmann S, Nahnsen S, Köster J. 2021. Sustainable data analysis with Snakemake. F1000Res 10:33.

2. Shindoh S, Kadoya A, Kanechi R, Watanabe K, Suzuki S. 2023. Marine bacteria harbor the sulfonamide resistance gene sul4 without mobile genetic elements. Front Microbiol 14.

3. Su R, Wen Y, Prabakusuma AS, Tang X, Huang A, Li L. 2023. Prevalence, antibiotic resistance and virulence feature of Listeria monocytogenes isolated from bovine milk in Yunnan, Southwest China. Int Dairy J 144.

4. Camacho C, Coulouris G, Avagyan V, Ma N, Papadopoulos J, Bealer K, Madden TL. 2009. BLAST+: architecture and applications. BMC Bioinformatics 10:421.

5. Katoh K, Misawa K, Kuma K-I, Miyata T. 2002. MAFFT: a novel method for rapid multiple sequence alignment based on fast Fourier transform. Nucleic Acids Res 30:3059–66.

6. Price MN, Dehal PS, Arkin AP. 2010. FastTree 2 - Approximately maximum-likelihood trees for large alignments. PLoS One 5.

7. Blanco-Míguez A, Beghini F, Cumbo F, McIver LJ, Thompson KN, Zolfo M, Manghi P, Dubois L, Huang KD, Thomas AM, Nickols WA, Piccinno G, Piperni E, Punčochář M, Valles-Colomer M, Tett A, Giordano F, Davies R, Wolf J, Berry SE, Spector TD, Franzosa EA, Pasolli E, Asnicar F, Huttenhower C, Segata N. 2023. Extending and improving metagenomic taxonomic profiling with uncharacterized species using MetaPhlAn 4. Nat Biotechnol 41:1633–1644.

8. McMurdie PJ, Holmes S. 2013. Phyloseq: An R Package for Reproducible Interactive Analysis and Graphics of Microbiome Census Data. PLoS One 8.

9. RStudio Team. Inc. Boston. 2016. Integrated development for R. RStudio. http://www.rstudio.com/.

10. Oksanen J, Blanchet FG, Friendly M, Kindt R, Legendre P, Mcglinn D, Minchin PR, O’hara RB, Simpson GL, Solymos P, Henry M, Stevens H, Szoecs E, Maintainer HW. 2020. vegan: Community Ecology Package.

11. Wickham H. 2016. ggplot2: Elegant graphics for data analysis. Springer-Verlag New York.

12. Li T, Zhang X, Luo F, Wu FX, Wang J. 2020. MultiMotifMaker: A multi-thread tool for identifying DNA methylation motifs from Pacbio reads. IEEE/ACM Trans Comput Biol Bioinform 17:220–225.

13. Veera Partanen A, Dekić Rozman S, Karkman A, Muurinen J, Hiltunen T, Virta M. 2025. Tracking horizontal gene transfer of antimicrobial resistance genes in microbial community with sequence barcodes. ISME J 5.

14. Schwengers O, Jelonek L, Dieckmann MA, Beyvers S, Blom J, Goesmann A. 2021. Bakta: Rapid and standardized annotation of bacterial genomes via alignment-free sequence identification. Microb Genom 7.

15. Shen W, Le S, Li Y, Hu F. 2016. SeqKit: A cross-platform and ultrafast toolkit for FASTA/Q file manipulation. PLoS One 11.

16. Fu L, Niu B, Zhu Z, Wu S, Li W. 2012. Sequence analysis CD-HIT: accelerated for clustering the next-generation sequencing data. Bioinformatics 28:3150–3152.

17. Hackl T, Trigodet F, Murat Eren A, Biller SJ, Eppley JM, Luo E, Burger A, DeLong EF, Fischer MG. 2021. proovframe: frameshit-correction for long-read (meta)genomics. bioRxiv https://doi.org/10.1101/2021.08.23.457338.

18. Buchfink B, Reuter K, Drost HG. 2021. Sensitive protein alignments at tree-of-life scale using DIAMOND. Nat Methods 18:366–368.

19. Gilchrist CLM, Chooi YH. 2021. Clinker & clustermap.js: Automatic generation of gene cluster comparison figures. Bioinformatics 37:2473–2475.

20. Yuan M, Nie L, Huang Z, Xu S, Qiu X, Han L, kang Y, Li F, Yao J, Li Q, Li H, Li D, Zhu X, Li Z. 2024. Capture of armA by a novel ISCR element, ISCR28. Int J Antimicrob Agents 64.

21. Madeira F, Madhusoodanan N, Lee J, Eusebi A, Niewielska A, Tivey ARN, Lopez R, Butcher S. 2024. The EMBL-EBI Job Dispatcher sequence analysis tools framework in 2024. Nucleic Acids Res 52:W521–W525.

22. Camargo AP, Roux S, Schulz F, Babinski M, Xu Y, Hu B, Chain PSG, Nayfach S, Kyrpides NC. 2023. Identification of mobile genetic elements with geNomad. Nat Biotechnol 42:1303–1312.

23. Roux S, Camargo AP, Coutinho FH, Dabdoub SM, Dutilh BE, Nayfach S, Tritt A. 2023. iPHoP: An integrated machine learning framework to maximize host prediction for metagenome-derived viruses of archaea and bacteria. PLoS Biol 21.

24. Yu MK, Fogarty EC, Eren AM. 2024. Diverse plasmid systems and their ecology across human gut metagenomes revealed by PlasX and MobMess. Nat Microbiol 9:830–847.

25. Murat Eren A, Kiefl E, Shaiber A, Veseli I, Miller SE, Schechter MS, Fink I, Pan JN, Yousef M, Fogarty EC, Trigodet F, Watson AR, Esen ÖC, Moore RM, Clayssen Q, Lee MD, Kivenson V, Graham ED, Merrill BD, Karkman A, Blankenberg D, Eppley JM, Sjödin A, Scott JJ, Vázquez-Campos X, McKay LJ, McDaniel EA, R Stevens SL, Anderson RE, Fuessel J, Fernandez-Guerra A, Maignien L, Delmont TO, Willis AD. 2021. Community-led, integrated, reproducible multi-omics with anvi’o. Nat Microbiol 6:3–6.

26. Bortolaia V, Kaas RS, Ruppe E, Roberts MC, Schwarz S, Cattoir V, Philippon A, Allesoe RL, Rebelo AR, Florensa AF, Fagelhauer L, Chakraborty T, Neumann B, Werner G, Bender JK, Stingl K, Nguyen M, Coppens J, Xavier BB, Malhotra-Kumar S, Westh H, Pinholt M, Anjum MF, Duggett NA, Kempf I, Nykäsenoja S, Olkkola S, Wieczorek K, Amaro A, Clemente L, Mossong J, Losch S, Ragimbeau C, Lund O, Aarestrup FM. 2020. ResFinder 4.0 for predictions of phenotypes from genotypes. J Antimicrob Chemother 75:3491–3500.

27. Langmead B, Salzberg SL. 2012. Fast gapped-read alignment with Bowtie 2. Nat Methods 9:357–359.

28. Chklovski A, Parks DH, Woodcroft BJ, Tyson GW. 2023. CheckM2: a rapid, scalable and accurate tool for assessing microbial genome quality using machine learning. Nat Methods 20:1203–1212.

29. Parks DH, Chuvochina M, Rinke C, Mussig AJ, Chaumeil PA, Hugenholtz P. 2022. GTDB: An ongoing census of bacterial and archaeal diversity through a phylogenetically consistent, rank normalized and complete genome-based taxonomy. Nucleic Acids Res 50:D785–D794.
