# Supplementary material for: Sulfonamide resistance gene sul4 is hosted by common wastewater sludge bacteria and found in various newly described contexts and hosts
Source: Microbiol Spectr. 2025 Nov 19;14(1):e00857-25. doi: 10.1128/spectrum.00857-25 (PMC12772301; doi:10.1128/spectrum.00857-25)
Supplement: Supplemental Material — Supplemental results and Figures S1 to S5. [file spectrum.00857-25-s0002.docx]

**Supplementary Results**

Sulfonamide resistance gene *sul4* is hosted by common wastewater sludge bacteria and found in various newly described contexts and hosts

Melina Markkanen^1,2^, Denise Pezzutto^1^, Marko Virta^1,2^, Antti Karkman^1,2^

^1^ Department of Microbiology, University of Helsinki, Helsinki, Finland

^2^ Multidisciplinary Center of Excellence in Antimicrobial Resistance Research, University of Helsinki, Helsinki, Finland

Contents:

Phylogenetic tree of *sul4* resembling genes

Supplementary Figure S1

Methylation method validation

Supplementary Figure S2

Contigs included in methylation analysis:

Supplementary Figure S3

Bacterial diversity by Metaphlan4:

Supplementary Figure S4

Phylogenetic analysis of ISCR elements

Supplementary Figure S5

*sul4* module of group I sequences were located in proximity to a prophage region

Phylogenetic tree of *sul4* resembling genes

Supplementary Figure S1. Phylogenetic tree of *sul* reference genes and *sul4* resembling genes. Established *sul* genes found in the ResFinder database (1) are marked in red. Sequence accession denoted in blue represent the suggested *sul4* genes carried by gram-positive bacteria described by Shindoh et al. 2023 and Su et al. 2023 (2, 3). Green labels indicate the genomic reference sequences gathered as a result of BLAST (4) search from the NCBI. The tree was rooted by the *fol*P gene of *Escherichia coli* K12 (NC_000913.3).

Methylation method validation

We tested our methylation-based approach with MultiMotifMaker (5) using a synthetic community with known bacterial composition by the available whole genome sequences of the community members (6). The methylation-based ordination of contigs showed that for certain community species, the clustering by methylation is strongly distinctive (e.g., *Pseudomonas_E chlororaphis*, *Pseudomonas_E putida*, *Paracoccus denitrificans, Enterococcus faecalis, Sphingobium yanoikuyae,* etc.) while for others, no species-wise distinctive clustering can be observed (e.g., *Sphingobacterium* sp.) (Supplementary Figure S2). Interestingly, certain species, such as *Citrobacter_B koseri,* seemed to possess sub-clustering within species (Supplementary Figure S2C), which could suggest that this method could possibly detect strain-level differences that emerge within a community during treatment. These species-wise differences in methylation detection sensitivity and specificity described above may have been caused either by biological or technical reasons. Examples of the first include heterogeneous or sparse methylation motifs due to horizontally acquired and lost methyltransferases (7). For the latter, technical factors such as low read coverage or short contig length may have contributed to the weak or undetected methylation signals (7). From these results, we concluded that methylation-based binning using MultiMotifMaker performs well in connecting metagenomic contigs into MAGs for a subset of species. Furthermore, for those species for which no MAGs could be binned, there seemed not to be a risk of reporting false results, as those contigs were either not showing any methylation profile or fell into the miscellaneous group of contigs with no form to be misidentified as a distinct cluster (Supplementary Figure S2). This gave us confidence to apply the method to shed light on the unknown carriers of *sul4*.

A B


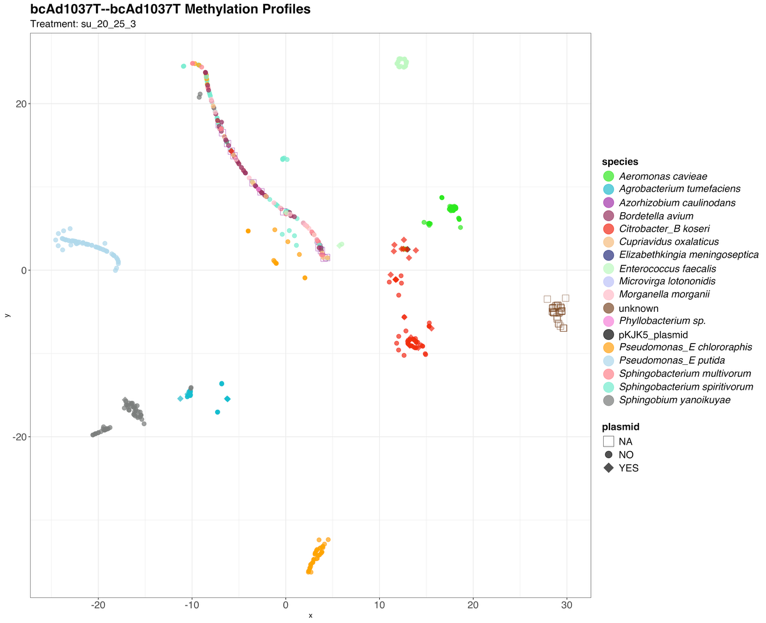

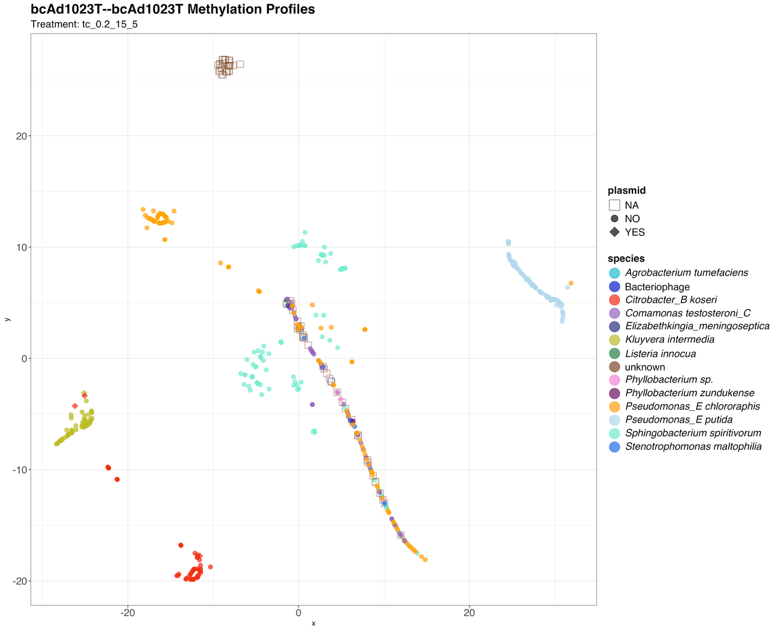


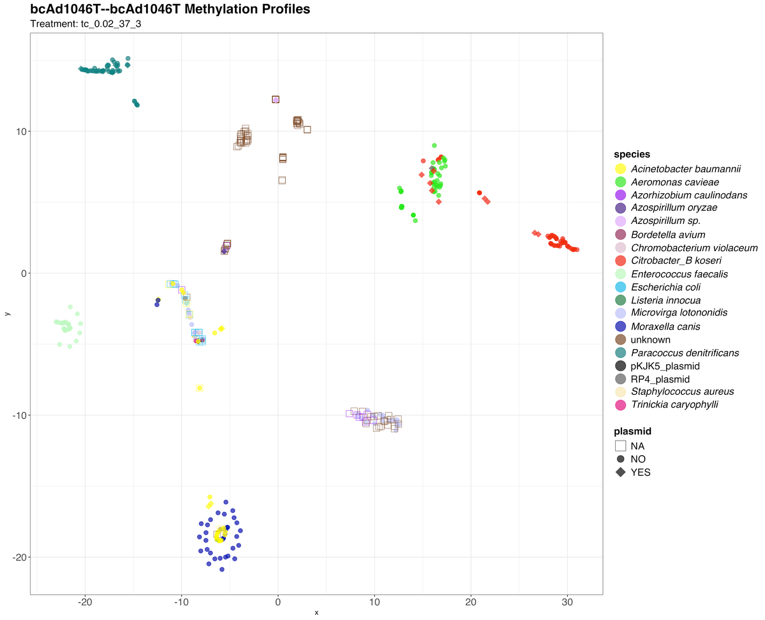
C D


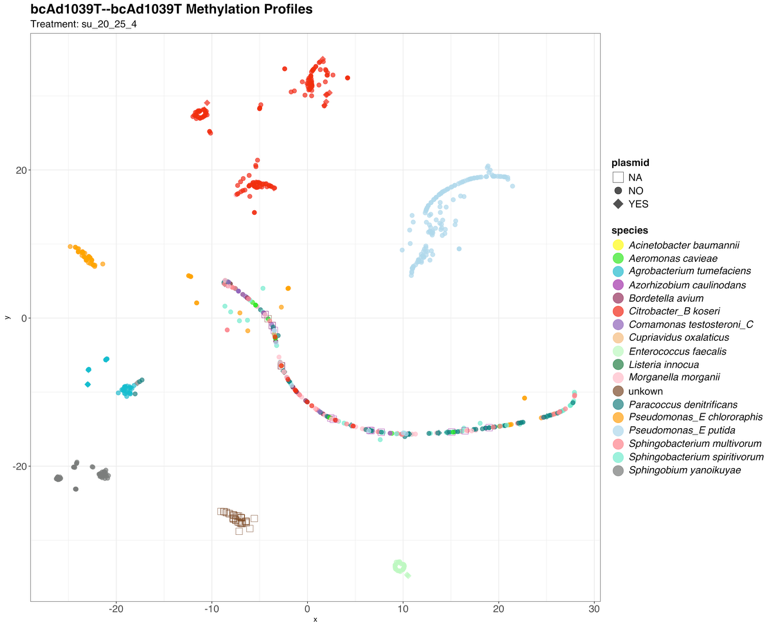


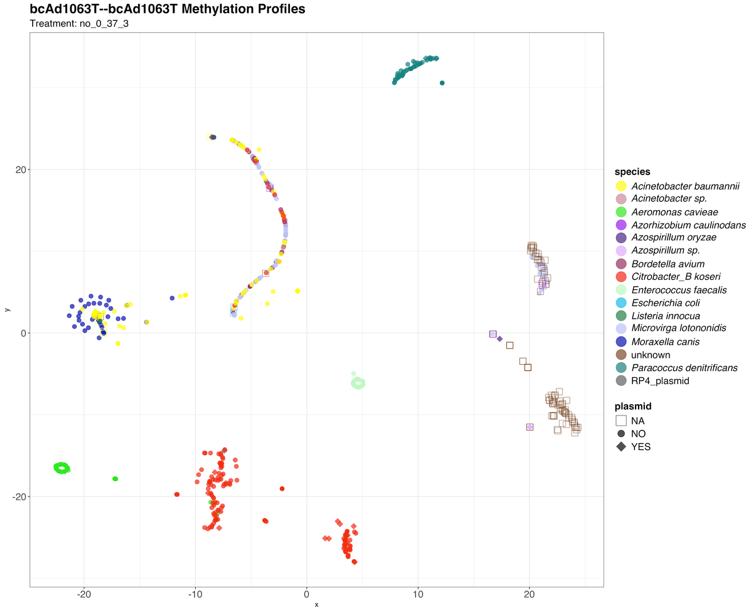
E

Supplementary Figure S2. t-SNE ordination of *de novo* predicted (MultiMotifMaker) (5) methylation profiles of metagenomic contigs of synthetic communities with known bacterial composition. Figures S2A-E represent distinct community samples with different treatments. Details of the synthetic community composition and sample treatments are described in Partanen et al. 2025.

Contigs included in methylation analysis


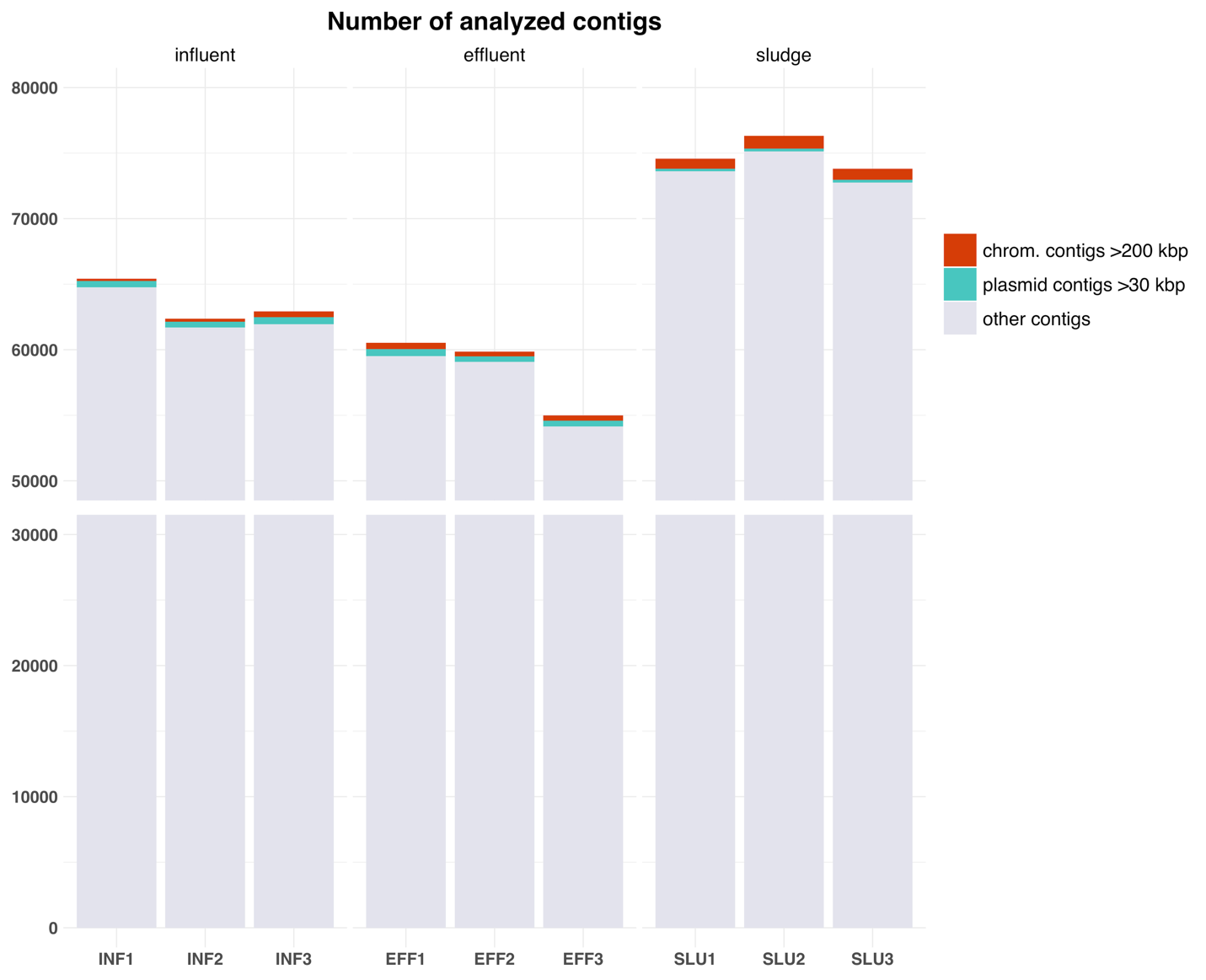


Supplementary Figure S3. Number of contigs explored for shared methylated motifs with *sul4* contigs

Bacterial diversity by Metaphlan4

Decreasing bacterial diversity was observed at genus level from influent towards the dried sludge. However interestingly, the trend was the opposite at phylum level likely reflecting the absence of typical well characterized human gut microbes from the class Gammaproteobacteria, for which conditions such as temperature have changed into less favorable in the sludge. On the other hand, more sludge-related environmental bacteria whose lower taxonomy level identities are not well recognized or captured by the taxonomical marker genes in the databases had possibly proliferated. Moreover, the Metaphlan4 database applies the former classification of Deltaproteobacteria in which Myxococcota and Desulfobacterota are not considered individual phyla as today.


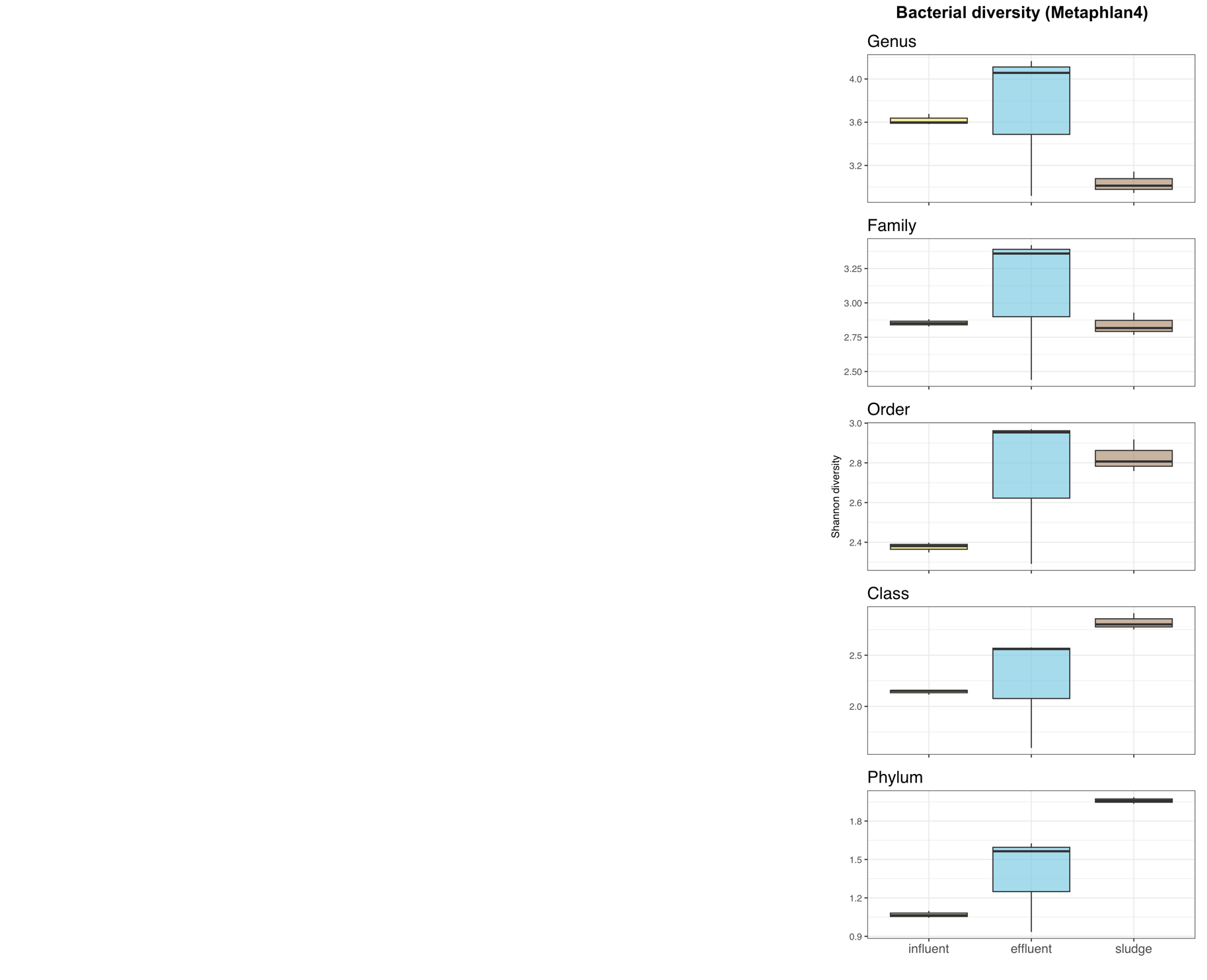


Supplementary Figure S4. Bacterial diversity by Shannon index calculated for influent, effluent and dried sludge at different taxa levels.

Phylogenetic analysis of ISCR elements


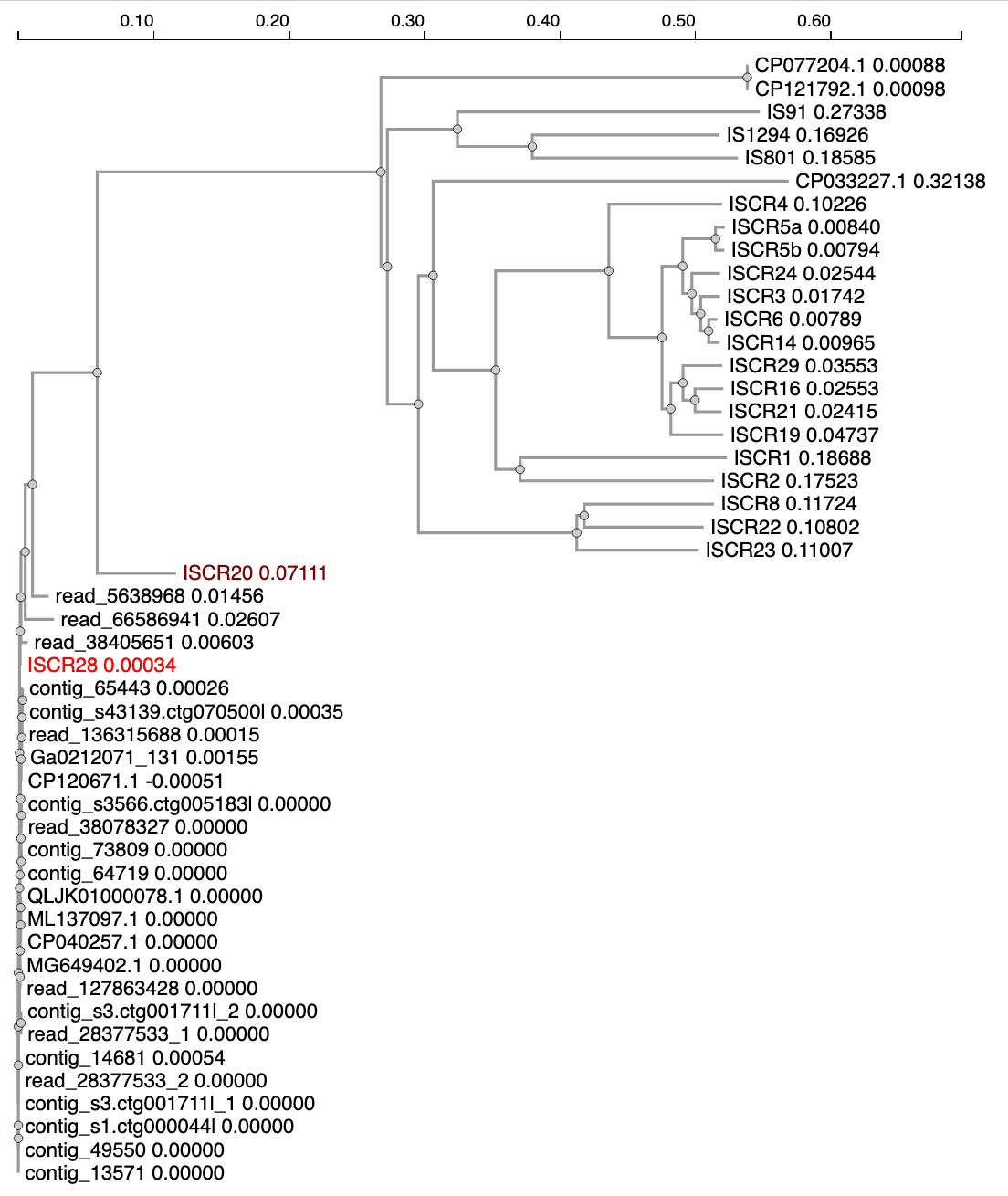


Supplementary Figure S5. Multiple sequence alignment of ISCR and canonical IS91 sequences from the study by Yuan et al. 2024 (8) (Table S4) and the ISCR elements of this study.

*sul4* module of group I sequences were located in proximity to a prophage region

To understand better the features of different wider contexts of the conserved *sul4* modules, the gene contents of 10 kb upstream and downstream *sul4* were investigated in more detail. Sequences categorized into group I based on their similar wider genetic contexts included *sul4* reads and contigs from sludge and effluent wastewater (Figure 1). Analysis with geNomad revealed that the conserved *sul4* module in these sequences was located adjacent to a prophage sequence region (Table S1). Depending on the length of the input sequence, the prophage region ranged between 8 702 and 56 197 bp in length and was located adjacent to the *sul4* or the integron integrase gene, extending upstream well beyond the area depicted in Figure 1. The average virus score for these regions defined by geNomad was 0.84 and the viral taxonomy was assigned as class Caudoviricetes (Table S1). Although genes related to prophage sequences were found also downstream *sul4* by Bakta, geNomad did not report this region as a prophage region. For the majority of proviral sequences with any assigned bacterial host taxa predicted by iPHoP (4/6), the host was determined as genus *Geobacter* (class Desulfuromonadia, phylum Desulfobacterota), with over 90% confidence value (Table S2). Various phage-related genes were detected among the group I sequences when considering the whole contig or read (Table S3). These included for example genes encoding phage major capsid protein (UniRef50_E1QHY6), capsid decoration protein (UniRef50_E1QHY7), and phage tail protein (UniRef50_Q310G1) (Table S3). However, the detection of these genes among the sequences somewhat varied even among those with otherwise similar sequences (Table S3).

Aiming to understand the putative significance of the prophage region for *sul4* module in Myxococcota genomes, non-*sul4* MAGs of *DUVQ01 sp012840965* were compared to *sul4* MAGs of *DUVQ01 sp012840965* for their viral sequence content. A prophage region was detected in one contig of the two non-*sul4* MAGs (SLU2_methylation). Additionally, the hypothetical protein-encoding genes within the 10 kb upstream *sul4* showed high similarity to those in the *sul4* contigs (Figure 1, Table 5). In a BLAST sequence comparison between the prophage sequence in the non-*sul4* contig (contig_s3.ctg003098l) and *sul4* contig (contig_s1.ctg000044l) with similar size prophage region (52,659 bp and 54,885 bp, respectively) the aligned sequence was 3,576 bp out of 4,711 bp, with an identity percentage of 76% (data not shown). Of the screened phage-related genes, two out of ten genes of *sul4* contig were present in the non-*sul4* contig (Table S3). The geNomad virus prediction score was lower for the non-*sul4* contig compared to most *sul4* contigs, despite the non-*sul4* contig having the highest number of viral genes (Table S1). Finally, the phage host prediction results by iPHoP differed from those of *sul4* contigs (Table S2).

The genetic context beyond the *sul4* module in the contig belonging to the Chloroflexota MAG was highly similar to those in Myxococcota *DUVQ01 sp012840965*, including the prophage region (Figure 1). In the Chloroflexota MAG contig, this prophage region was only 8,702 pb in length. However, when the entire contig was considered, it shared five of the ten screened phage-related genes found in contig_s1.ctg000044l (Table S3).

References:

1. Bortolaia V, Kaas RS, Ruppe E, Roberts MC, Schwarz S, Cattoir V, Philippon A, Allesoe RL, Rebelo AR, Florensa AF, Fagelhauer L, Chakraborty T, Neumann B, Werner G, Bender JK, Stingl K, Nguyen M, Coppens J, Xavier BB, Malhotra-Kumar S, Westh H, Pinholt M, Anjum MF, Duggett NA, Kempf I, Nykäsenoja S, Olkkola S, Wieczorek K, Amaro A, Clemente L, Mossong J, Losch S, Ragimbeau C, Lund O, Aarestrup FM. 2020. ResFinder 4.0 for predictions of phenotypes from genotypes. J Antimicrob Chemother 75:3491–3500.

2. Shindoh S, Kadoya A, Kanechi R, Watanabe K, Suzuki S. 2023. Marine bacteria harbor the sulfonamide resistance gene sul4 without mobile genetic elements. Front Microbiol 14.

3. Su R, Wen Y, Prabakusuma AS, Tang X, Huang A, Li L. 2023. Prevalence, antibiotic resistance and virulence feature of Listeria monocytogenes isolated from bovine milk in Yunnan, Southwest China. Int Dairy J 144.

4. Camacho C, Coulouris G, Avagyan V, Ma N, Papadopoulos J, Bealer K, Madden TL. 2009. BLAST+: architecture and applications https://doi.org/10.1186/1471-2105-10-421.

5. Li T, Zhang X, Luo F, Wu FX, Wang J. 2020. MultiMotifMaker: A multi-thread tool for identifying DNA methylation motifs from Pacbio reads. IEEE/ACM Trans Comput Biol Bioinform 17:220–225.

6. Veera Partanen A, Dekić Rozman S, Karkman A, Muurinen J, Hiltunen T, Virta M. 2025. Tracking horizontal gene transfer of antimicrobial resistance genes in microbial community with sequence barcodes. ISME J 5.

7. Beaulaurier J, Zhu S, Deikus G, Mogno I, Zhang XS, Davis-Richardson A, Canepa R, Triplett EW, Faith JJ, Sebra R, Schadt EE, Fang G. 2018. Metagenomic binning and association of plasmids with bacterial host genomes using DNA methylation. Nat Biotechnol 36:61–69.

8. Yuan M, Nie L, Huang Z, Xu S, Qiu X, Han L, kang Y, Li F, Yao J, Li Q, Li H, Li D, Zhu X, Li Z. 2024. Capture of armA by a novel ISCR element, ISCR28. Int J Antimicrob Agents 64.
